# Transcriptome Sequencing and Expression Analysis of Cadmium (Cd) Transport and Detoxification Related Genes in Cd-Accumulating *Salix integra*

**DOI:** 10.3389/fpls.2016.01577

**Published:** 2016-10-28

**Authors:** Xiang Shi, Haijing Sun, Yitai Chen, Hongwei Pan, Shufeng Wang

**Affiliations:** ^1^Research Institute of Subtropical Forestry, Chinese Academy of ForestryHangzhou, China; ^2^Key Laboratory of Tree Breeding of Zhejiang ProvinceHangzhou, China

**Keywords:** willow, *Salix integra*, Cd stress, transcription factor, *de novo* assembly, Cd transportation

## Abstract

*Salix integra* is a shrub willow native to northeastern China, Japan, Korea, and Primorsky Krai in the far southeast of Russia, and has been identified as cadmium (Cd)-accumulating trees in recent years. Although many physiological studies have been conducted with these plants, little is known about the molecular basis underlying Cd response in this plant, and this is confirmed by the very few number of gene sequences (only 39 nucleotide sequences) available in public databases. Advances in genomics for *Salix* are promising for future improvement in identification of new candidate genes involved in metal tolerance and accumulation. Thus, high-throughput transcriptome sequencing is essential for generating enormous transcript sequences from *S. integra*, especially for the purpose of Cd toxicity-responsive genes discovery. Using Illumina paired-end sequencing, approximately 60.05 million high-quality reads were obtained. *De novo* assembly yielded 80,105 unigenes with an average length of 703 bp, A total of 50,221 (63%) unigenes were further functionally annotated by comparing their sequences to different proteins and functional domain databases. GO annotation reveals 1849 Cd responsive genes involving in Cd binding, transport, and detoxification and cellular Cd homeostasis, and these genes were highly enriched in plant response to Cd ion and Cd ion transport. By searching against the PlantCyc database, 509 unigenes were assigned to 14 PlantCyc pathways related to Cd transport and cellular detoxification, and many of them are genes encoding heavy metal ATPases (*HMA*s), nature resistance-associated with microphage proteins (*NRAMP*s), ATP-binding cassette (ABC) transporters, etc., Comprehensive RT-qPCR analysis of these selected genes in different tissues of *S. integra* under the control and Cd treatment revealed metallothionein-like protein (*MT2A* and *MT2B*), Metal tolerance protein (*MTP1*), *ABCB25, NRAMP5*, and *ZIP1* may be involved in the Cd transport and detoxification in leaves, while *NRAMP2, ZIP8*, and *NRAMP5* may be related to Cd transport in roots. Our study will enrich the sequence information of *S. integra* in public database, and would provide some new understanding of the molecular mechanisms of heavy metal tolerance and detoxification in willows.

## Introduction

Cadmium (Cd) is known to be one of the most toxic non-essential elements in soil. It is very mobile and easily accumulates in plant tissues, and therefore readily enters into food chain, which cause potential threat to human health with the high risk of causing cancer (Nawrot et al., 2006; Satarug et al., 2010). A promising approach to remediate Cd contamination is phytoextraction, the use of plants to clean up polluted areas (Salt et al., 1995). Efficient phytoextraction species should translocate high quantities of heavy metals (HMs) into their aboveground biomass without toxicity symptoms, and at the same time produce large amounts of biomass (Vangronsveld et al., 2009; Luo et al., 2016).

Willow species (*Salix* spp.) have been used for phytoremediation of heavy metal contaminated areas because of their fast growth, large biomass production, and adaptation to many nutrient-poor or wet conditions (Dos Santos Utmazian et al., 2007; Kuzovkina and Volk, 2009; Marmiroli et al., 2011). *Salix* could accumulate considerable amounts of Cd in their aboveground organs, for example, the goat willow (*Salix caprea*) accumulated up to 116 mg Cd kg^−1^ dry weight in leaves in a metal-polluted habitat (Dos Santos Utmazian et al., 2007). *Salix integra* is a fast-growing shrub willow, and has been identified as Cd-accumulating trees at old mining sites (Nagashima et al., 2005). In China, it is generally cultivated by short-rotation plantation, with the new shoots harvested for use in weaving. Some of the cultivated varieties, such as Yizhibi and Weishanhu, displayed excellent in biomass production and environmental adaptation. They could accumulate up to 288 mg Cd kg^−1^ dry weight in their leaves, and more interestingly, *S. integra* was shown to be able to grow in solutions with 90 μmol/L of Cd concentration with moderate symptoms of toxicity (Wang et al., 2013). This means *S. integra* could not only accumulate desired Cd in the aboveground tissues, but also have high tolerance to Cd in their roots. Although the metal-accumulating ability of *S. integra* has been well documented, basic understanding of molecular mechanisms of metal tolerance, and accumulation is very limited.

Concerning heavy metals tolerance and accumulation in plants, many genes involved in adaptive tolerance and detoxification of HMs have been identified and isolated from several plants (Verbruggen et al., 2009; Krämer, 2010; Ueno et al., 2011; Luo et al., 2016). A network of transporters related to metal uptake into roots, xylem loading, and vacuolar sequestration (Broadley et al., 2007; Verbruggen et al., 2009; Pottier et al., 2015; Song et al., 2016) were discovered in model plants and some hyperaccumulators. It is reported that HMs are taken up by root cells via plasma membrane-located transporters, like nature resistance-associated with microphage proteins (*NRAMPs*) and zinc/iron regulated proteins (*ZIPs*) etc., and heavy metal ATPases (*HMAs*), Metal tolerance proteins (*MTPs*), ATP-binding cassette (ABC) transporters play a key role in cellular metal efflux in most herbaceous plants (Dräger et al., 2004; Hanikenne et al., 2008; Gao et al., 2013; Li et al., 2015; Pottier et al., 2015). In *Arabidopsis thaliana*, metallothioneins (*MT*s), and phytochelatin synthases(*PC*s) work cooperatively to mediate Cu and Cd toxicity (Guo et al., 2008). In general, plants have evolved several strategies of HM sequestration and detoxification to reduce the toxicity of HMs.

Woody plants could have more complicated mechanisms, which requires broad scale transcriptional, proteomic, and metabolic approaches. However, limited information is available about molecular mechanisms of HM tolerance and detoxification in trees, and most of HM related genes in woody plants are restricted to *Populus* (Luo et al., 2016). The lack of genomic information for woody plants has hindered the understanding of molecular mechanism of HM tolerance and detoxification. In recent years, the high-throughput next generation sequencing (NGS) technology such as RNA-seq has become an effective tool to discover molecular markers and identify novel genes, and has been successfully used for studying plants without genomic sequence (Wang et al., 2009, 2010). However, only a few studies on transcriptional responses of *Salix* to Cd contamination (Gouker, 2013; Yang J. L. et al., 2015) were reported, especially the public available data for *S. integra* are far from sufficient to understand the molecular basis involved in metal tolerance and detoxification, with only 39 nucleotide sequences from *S. integra* deposited in the public databases.

In the present study, we utilized the Illumina paired-end sequencing technology to characterize the transcriptome of *S. integra*. Our aim was to get the general transcriptomic information of *S. integra*, and to identify the Cd transportation and detoxification related genes based on the transcriptome sequence annotation and RT-qPCR analysis. To maximize the diversity of transcriptional units, a pooled RNA collection from different tissues and different treatments were used to generate a broad survey of genes associated with willow growth and development, as well as environmental response. We hypothesized that *S. integra* may possess similar molecular mechanisms of HM tolerance and detoxification with herbaceous, and some metal related genes characterized in model plants or *Populus* could also function in *S. integra*. Based on the assumption that the putative transporters and metal-chelation proteins may involve in Cd transmembrane transport and cellular detoxification in *S. integra*, we also hope to predict the function of selected candidate genes among different organs. To the best of our knowledge, this study is the first exploration to characterize the transcriptome of *S. integra* using high-throughput sequencing platform, and the transcriptome sequencing from *S. integra* will help in furthering our understanding of the molecular mechanisms of heavy metal adaptation in willows.

## Materials and methods

### Plant material and RNA extraction

The *S. integra* were obtained from native habitats in Shandong Province. No specific permits were required to extract samples from this site, which is not privately owned, and this study did not involve protected species. Cuttings (8–10 cm) from 1-year-old stems in nursery beds at the Institute of Subtropical Forestry were selected for uniformity based on the diameter (Φ 0.4–0.5 cm) and the number of buds (4–6 buds per cutting). Cuttings were rooted in tap water for 4 weeks in hydroponic pots (50 × 35 cm × 15 cm, length × width × height) and then transferred to 15 L aerated Knop's solution (Magdziak et al., 2011) (6.1 mM Ca (NO_3_)_2_, 2.5 mM KNO_3_, 1.6 mM KCl, 1.8 mM KH_2_PO_4_, 2.1 mM MgSO_4_, and 3.8 μM FeCl_3_) in each pot, maintaining a constant pH of 5.5 using 1 M HCl, or 1 M NaOH.

After 2 weeks of plant growth in the aerated solution, Cd treatment with the final concentration of 50 μM was applied as Cd (NO_3_)_2_ for 24 h, with normal nutrient solution as the control. RNA was extracted separately from the leaves, stems, and roots of the control and of the treated *S. integra*. Total RNA extraction was perfomed by using the RN38 EASYspin plus Plant RNA kit (Aidlab Biotech, Beijing, China) following the manufacturer's instructions. RNA concentration and integrity were analysized using Nanodrop 2000 (Thermo Scientific, USA) and Agilent 2100 Bioanalyzer (Agilent Technologies, Santa Clara, CA, USA) respectively. A total of 2.0 μg of RNA was equally pooled from the three tissues of the control and the treatment for cDNA library preparation. The experiment was performed with three repetitions, each repetition included the control, and the treatment with five plants as biological replicates respectively, and used for both RNA-seq and quantitative real time PCR analysis.

### cDNA library construction and RNA-seq

The mRNA-seq library was constructed using Illumina's TruSeq RNA Sample Preparation Kit (Illumina Inc, San Diego, CA, USA). The isolation of mRNA, fragment interruption, cDNA synthesis, addition of adapters, PCR amplification and RNA-Seq were performed by BioMarker Technologies (Beijing, China). Poly-A mRNA was isolated using poly-T oligo-attached magnetic beads, and then broken into small pieces using divalent cations under an elevated temperature. The cleaved RNA fragments were copied into first strand cDNA using reverse transcriptase and random primers. This was followed by second strand cDNA synthesis using DNA Polymerase I and RNase H. A single “A” base was ligated to the short fragments after being purified using a MinElute PCR Purification Kit (Qiagen, Dusseldorf, Germany), preparing them for ligation to the sequencing adapters. Fragments (200 ± 25 bp) were then separated by agarose gel electrophoresis and selected for PCR amplification as sequencing templates. Finally, the mRNA-seq library was constructed for sequencing on the Illumina HiSeqTM 2000 sequencing platform, and 2 × 101 bp reads were yielded from either end of a cDNA fragment.

### Sequence data analysis and assembly

To obtain high-quality clean read data for *de novo* assembly, the raw reads from RNA-seq were filtered by removing the reads with adaptor contamination, low-quality reads with ambiguous “N” bases, and reads in which more than 10% bases had a *Q* < 20 (Wang et al., 2010; González and Joly, 2013). All sequences smaller than 60 bases were eliminated based on the assumption that small reads might represent sequencing artifacts. The quality reads were assembled into unigenes with Trinity which recovers more full length transcripts across a broad range of expression levels, with sensitivity similar to methods that rely on genome alignments (Grabherr et al., 2011). The overlap settings used for this assembly were 31 bp and 80% similarity, with all other parameters set to their default values.

### Sequence annotation

To determine the functional annotation of the unigenes, a BLASTX search was performed against the NCBI Nr database with an *E* ≤ 10^−5^ and other databases, including SwissProt, Protein Information Resource (PIR), Protein Research Foundation (PRF), and Protein Data Bank (PDB). Gene names were assigned based on the annotation of the closest UniProt match, with uninformative descriptions excluded. A BLASTN search was also performed against the NCBI Nt database, GenBank, RefSeq, and PDB using a protein query. The ORFs were identified as the nucleotide sequence or as the protein translation provided by the “GetORF” program from the EMBOSS software package (Rice et al., 2000). The longest ORF was extracted for each unigene. The expression abundance of the unigenes was represented by the number of reads per kilobase of exon model per million mapped reads (RPKM) (Mortazavi et al., 2008). The Blast2GO program was used to assign GO terms with an *E* ≤ 10^−5^ including molecular functions, biological processes, and cellular components (Conesa et al., 2005).

The unigenes sequences were also aligned to the COG database to predict and classify functions. KEGG pathways were retrieved from KEGG web server (http://www.genome.jp/kegg/). The output of the KEGG analysis includes KO assignments and KEGG pathways that are populated with the KO assignments. The metabolic pathways were annotated by PlantCyc Enzymes database v7.0 (www.plantcyc.org).

### Detection of SSR markers

The assembled sequences longer than 1 kb were used for the detection of SSR markers. Potential SSR markers were detected among the 5473 unigenes using MISA software (http://pgrc.ipk-gatersleben.de/misa/). The parameters were designed for the identification of perfect dinucleotide motifs with a minimum of six repeats, and tri-, tetra-, penta-, and hexa-nucleotide motifs with a minimum of five repeats (Zeng et al., 2010).

### Quantitative real-time PCR analysis

The *S. integra* transcriptome database was mined for genes involved in Cd transportation and detoxification pathways. The expression profiles include the genes involved P-type metal ATPase *HMA1, HMA2*, and *HMA5*, NRAMP family genes *NRAMP2, NRAMP5*, and *NRAMP6*, zinc transporter *ZIP1* and *ZIP8*, metal tolerance protein *MTP1*, metallothionein-like protein *MT2A* and *MT2B*, glutathione gamma-glutamylcysteinyl transferase1 *PCS1* and ABC transporter family member *ABCB25, ABCG36*, and *ABCG35*. cDNA synthesis was performed with 3 μg total RNA from leaves, stems and roots using the Superscript III First Strand Synthesis system followed by the RNase H step (Invitrogen, Carlsbad, USA), according to the manufacturer's protocol in a total volume of 20 μl. Primer pairs were designed using Primer3 (http://frodo.wi.mit.edu/primer3/) with the following parameters: Tm of approximately 60°C, product size range of 120–200 base pairs, primer sequences with a length of 20 nucleotides, and a GC content of 45–55%. The gene names, sequences and the primers used for RT-qPCR analysis are listed in File S1. To quantify the expression level, the selected genes, the *S. integra* ACT gene was used as an endogenous control. RT-qPCR reactions were performed in 96-well plates using a 7300 Real Time PCR System (Applied Biosystems, CA, USA) and a SYBR® Premix Ex Taq™ Kit (TaKaRa, Dalian, China). PCR reactions were prepared in 20 μl volumes containing 2 μl of 30-fold diluted synthesized cDNA, 10 μl 2 × SYBR® Premix Ex TaqTM, 0.4 μl 10 μM forward primer, 0.4 μl 10 μM reverse primer, 0.4 μl 50 × RO × reference dye and 6.8 μl sterile distilled water. The cycling conditions were recommended by the manufacturer (30 s at 95°C, 40 cycles of 95°C for 5 s, and 60°C for 31 s). The specificity of the amplicons was verified by melting curve analysis (60–95°C) after 40 PCR cycles. Three biological replicates (nested with three technical replicates) per sample were carried out, and the data analysis was performed as described by Pfaffl (2001).

## Results

### Investigation of transcriptome of *S. integra*

*S. integra* can accumulate more than 3000 mg kg^−1^ and 200 mg kg^−1^ DW of Cd in roots and the above ground parts respectively when grown in hydroponical solution in our previous study (Wang et al., 2013). To maximize the range of transcript diversity, a mixed RNA sample from three tissues and two Cd treatments was prepared for RNA-seq using the Illumina HiSeqTM 2000. Altogether, 12.13 gigabase pairs (Gbp) with an average GC content of 45.44% (Table 1) and 60.05 million high-quality reads were obtained. We defined the reads with *Q* ≥ 20 and no ambiguous “N” as high-quality reads. After primers and adaptors were removed, 60.05 million high-quality reads were assembled into 149,361 transcripts with a mean length of 1,150.47 bp using the Trinity *de novo* assembly program. The transcripts were subjected to cluster and assembly analyses. Finally, a total number of 80,105 unigenes were obtained with an average length of 703 bp, which included 15,007 unigenes (18.74%) with lengths greater than 1 kb. These results showed the throughput and sequencing quality was high enough for the following analyses. This transcriptome assembly project has been deposited at GenBank under the accession GEYA00000000.

**Table 1:** **Summary of Illumina transcriptome sequencing for *S. integra***.

**Sample ID**	**Total reads**	**Total nucleotides (bp)**	**GC (%)**	**N (%)**	**Q20 (%)**
*S. integra*	60,047,711	12,128,689,157	45.44	0.17	90.11

**Figure 1:**
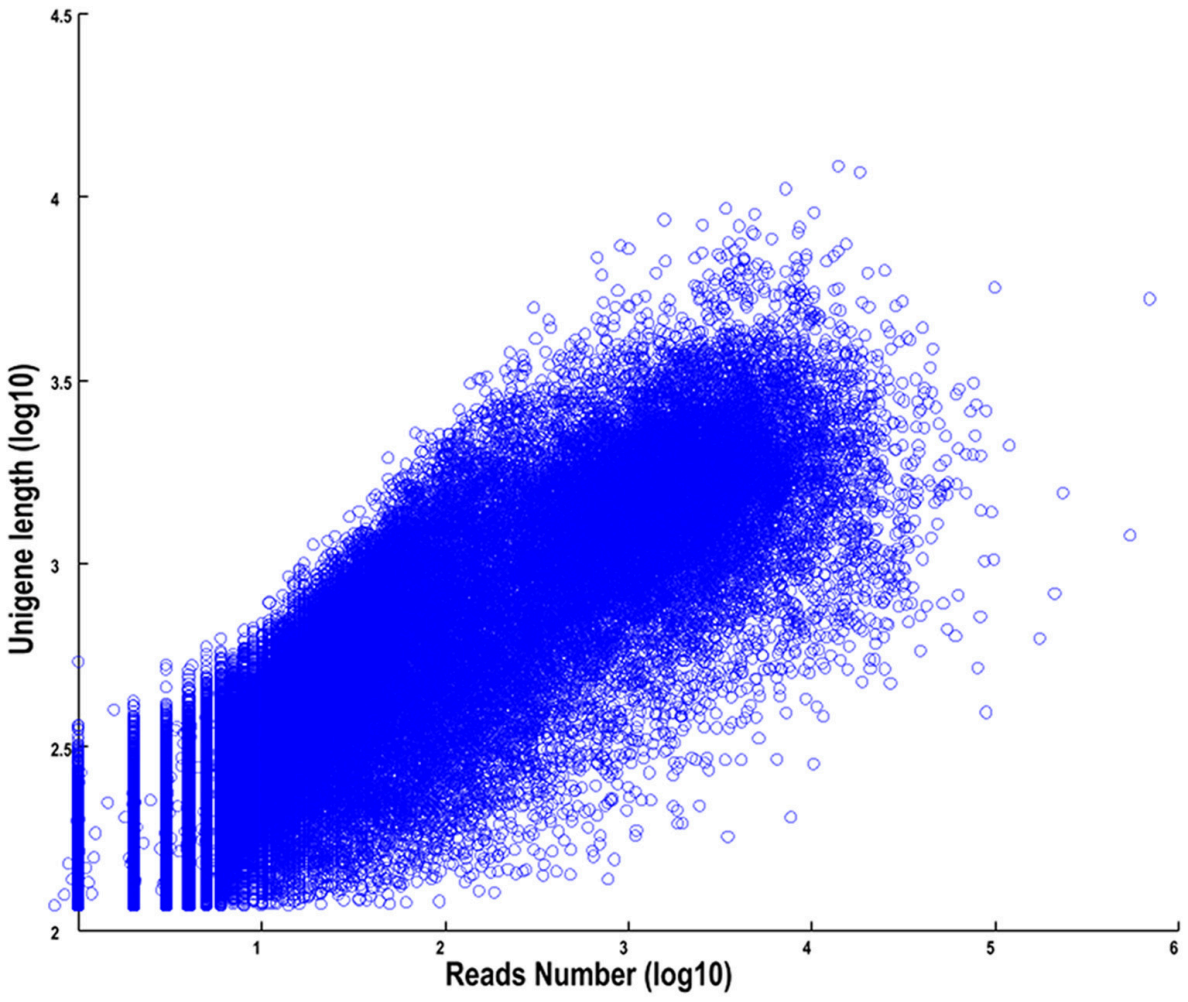
**The dependence of unigene lengths on the number of reads assembled into each unigene**.

The length distributions of transcripts and unigenes are listed in Table 2, which showed that the distribution of transcripts are similar with that of the unigenes. The N50 values of transcripts and unigenes were 1971 and 1191 bp, respectively. As expected for a randomly fragmented transcriptome, there was a positive relationship between the length of a given unigene and the number of reads (Figure 1). In addition, we made an Open Reading Frame (ORF) prediction analysis. ORFs were predicted using Getorf from the EMBOSS package and most unigenes (99.54%) were identified as having ORFs starting at an “ATG” codon. To facilitate the access and use of the *S. integra* transcriptome sequencing data, the raw paired-end sequence data in the FASTQ format was deposited in the National Centre for Biotechnology Information (NCBI) Sequence Read Archive (SRA) database with accession number SRR2155573.

**Table 2:** **Length distribution of assembled transcripts and unigenes**.

**Length_span**	**Numbers**	
	**Transcripts**	**Unigenes**
0–100	0	0
100–200	0	0
200–300	31,775	27,828
300–400	18,250	14,974
400–500	10,899	7976
500–600	7646	4897
600–700	6207	3421
700–800	5180	2477
800–900	4841	2012
900–1000	4158	1513
1000–1100	4047	1377
1100–1200	3816	1185
1200–1300	3688	986
1300–1400	3521	956
1400–1500	3372	878
1500–1600	3239	804
1600–1700	3109	779
1700–1800	2890	774
1800–1900	2702	685
1900–2000	2620	658
2000–2100	2326	550
2100–2200	2216	563
2200–2300	2017	477
2300–2400	1883	444
2400–2500	1736	389
2500–2600	1502	301
2600–2700	1423	309
2700–2800	1282	277
2800–2900	1146	252
2900–3000	1106	204
>3000	10,764	2159
Total number	14,9361	80,105
Total length	171,835,806	56,285,368
N50 length	1971	1191
Mean length	1,150.473055	702.6449

### Functional annotation of *S. integra* transcriptome classification

**Figure 2:**
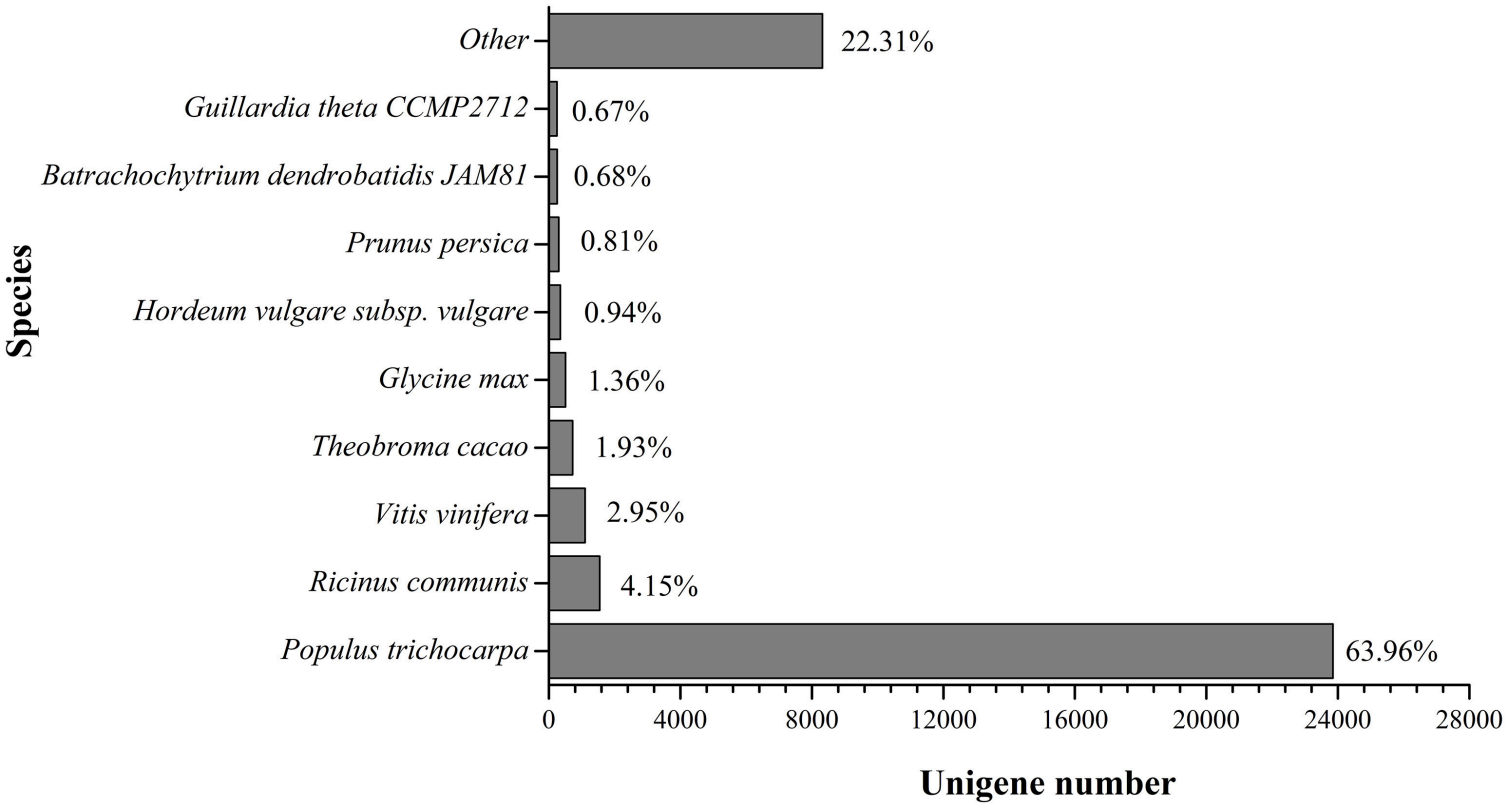
**Species distribution in BLAST hits in Nr dababase**. 36,041 BLASTX-hit unigenes were calculated.

Based on the alignments of willow unigenes to the databases, including the NCBI non-redundant protein (Nr) database, NCBI non-redundant nucleotide sequence (Nt) database, UniProt/Swiss-Prot, Kyoto Encyclopedia of Genes and Genomes (KEGG), Cluster of Orthologous Groups of proteins (COG) and UniProt/TrEMBL database, a total of 50, 221 annotative unigenes (62.7%) were obtained. The overall functional annotation for *S. integra* is listed in Table 3. According to the results, 41,823 (52.2%) unigenes had significant matches in the Nt database, and 29,793 (37.2%) unigenes had similarity to proteins in the Swiss-Prot database. Furthermore, 37,283 (46.5%) unigenes have homologous proteins in the Nr protein database, among which 63.96% (23,846) unigenes showed significant homology with sequences of *Populus trichocarpa* (Figure 2). Only 4.15% and 2.95% of the mapped sequences have a high similarity with sequences of *Ricinus communis* and *Vitis vinifera*, respectively.

**Table 3:** **Functional annotation of the unigenes of *S. integra***.

**Annoted_database**	**Annotated_number**	**Percentage of unigenes (%)**
COG_annotation	12,221	15.3
GO_annotation	32,570	40.7
KEGG_annotation	10,552	13.2
Swissprot_annotation	29,793	37.2
TrEMBL_annotation	36,923	46.1
Nr_annotation	37,283	46.5
Nt_annotation	41,823	52.2
All_annotated	50,221	62.7

**Figure 3:**
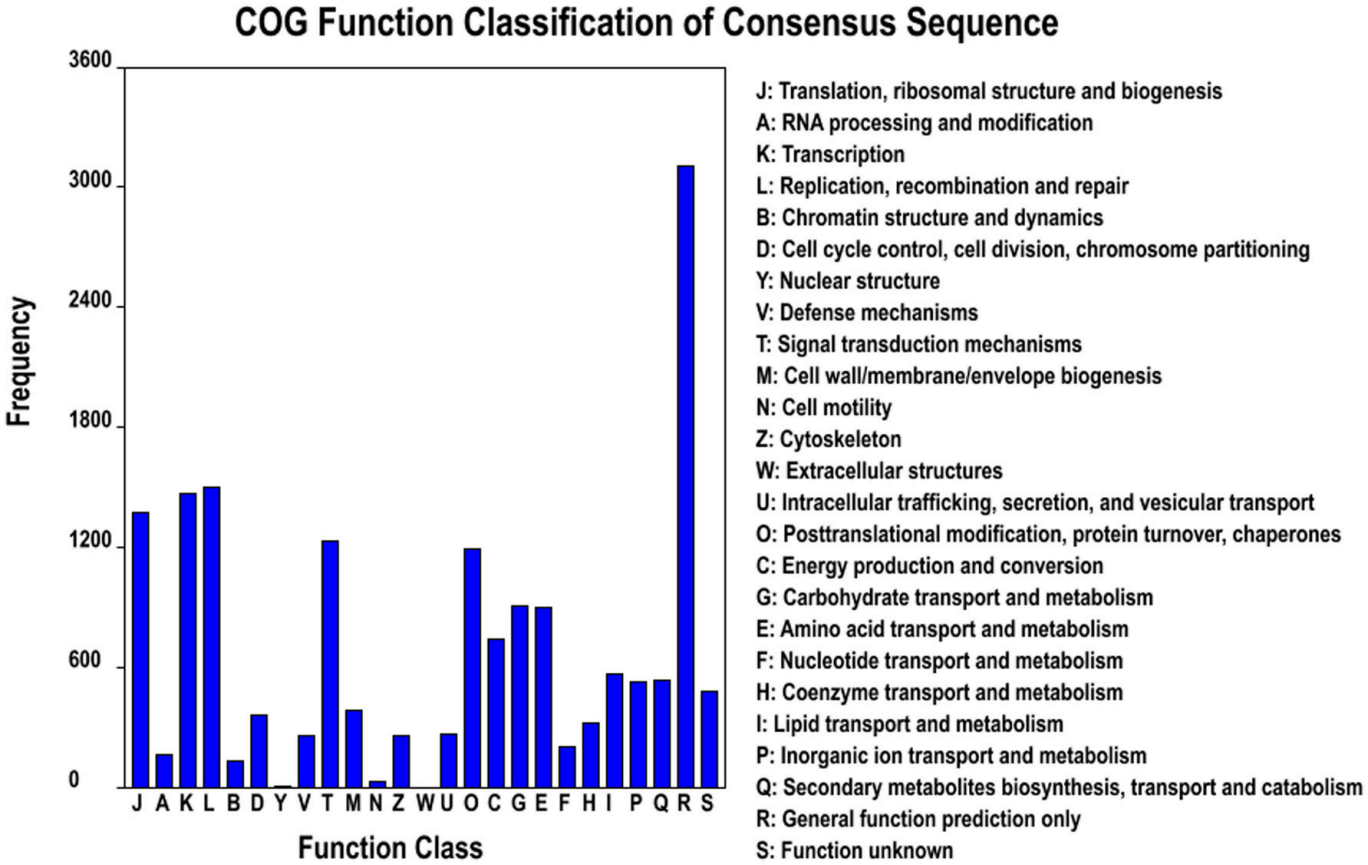
**Clusters of orthologous groups (COG) classification**. In total, 12,221 of the 80,105 unigenes with Nr hits were grouped into 25 classifications.

Based on the Nr annotation, 16,879 unigenes (33.6%) could be aligned to the COG database to predict and classify possible functions. According to the annotation of COG, these genes were classified into 25 different functional classes, including RNA processing and modification, energy production, and conversion, carbohydrate transport and metabolism, signal transduction mechanisms, lipid transport and metabolism, coenzyme transport and metabolism (Figure 3). The cluster for general function prediction (3105; 18.4%) represented the largest group, followed by replication, recombination and repair (1497; 8.87%), transcription (1469; 8.7%), translation, ribosomal structure, and biogenesis (1370; 8.12%), signal transduction mechanisms (1229; 7.28%), posttranslational modification, protein turnover, and chaperones (1197; 7.07%), carbohydrate transport and metabolism (908; 5.38%), amino acid transport and metabolism (897; 5.31%), and energy production and conversion (743; 4.4%). However, only a few unigenes were assigned to cell motility and nuclear structure (25 and 6 unigenes, respectively). In addition, no unigene was assigned to extracellular structures. Furthermore, 523 (3.1%) unigenes were assigned to inorganic ion transport and metabolism and 256 (1.52%) unigenes were assigned to defense mechanisms (Figure 3), and we found most of unigenes involved in Cd tolerance and detoxification were found in these two categories.

**Figure 4:**
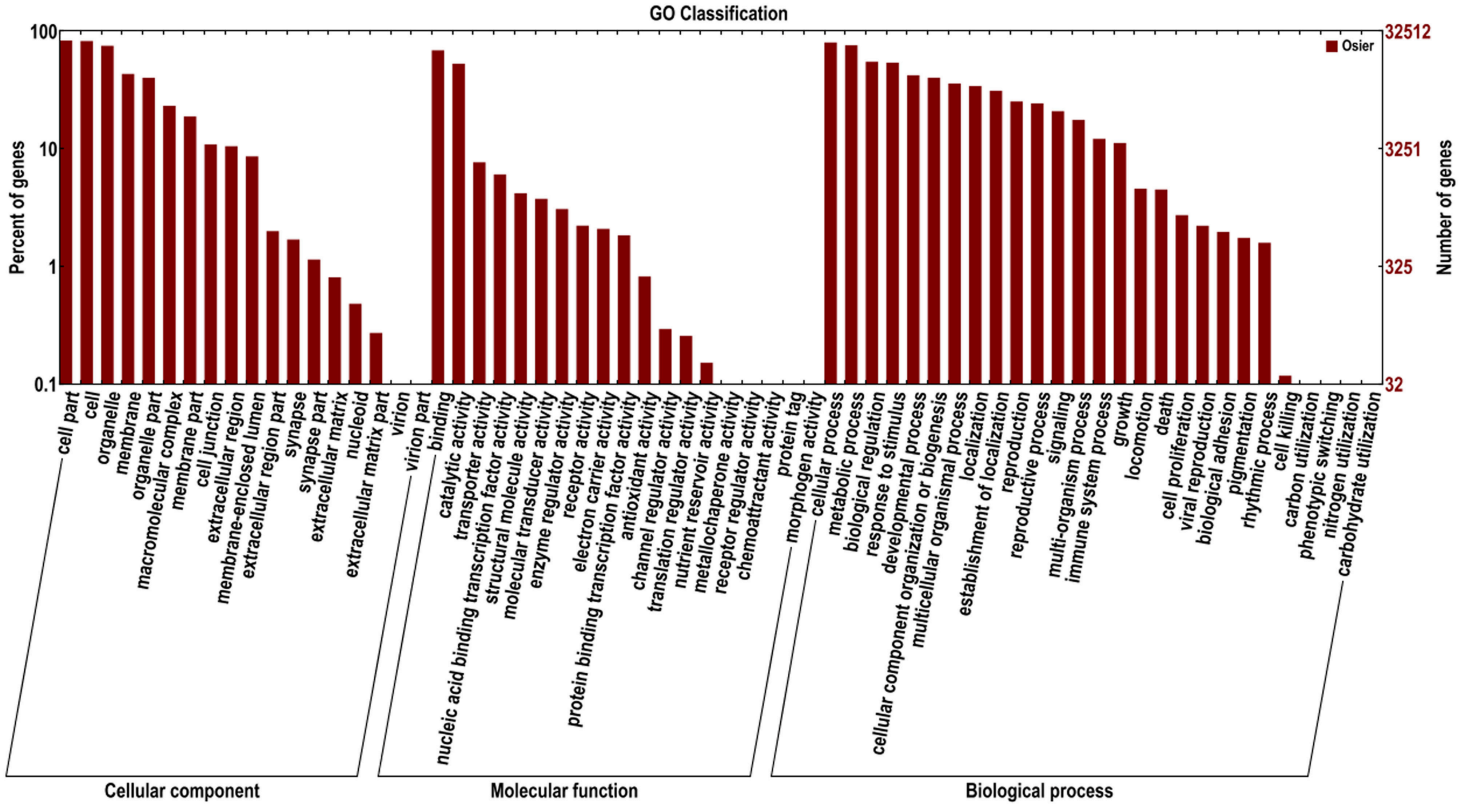
**Functional annotation of assembled sequences based on gene ontology (GO) categorization**. The unigenes are summarized into three main categories: cellular component, molecular function and biological process.

Functions of 32,570 (40.7%) unigenes were further classified by GO analysis, and were distributed into the three categories with 50.98% in biological processes, 35.44% in cellular components, and 13.57% in molecular functions (Figure 4). According to GO annotation, the three largest GO terms found in the “biological process” ontology were “cellular process,” “metabolic process” and response to stimulus, comprising 79.8, 75.5, and 53.7% of the GO- termed unigenes, respectively. In the “cellular component” and “molecular function” ontologies, the top terms were “cell part” and “binding,” which are 82.9 and 68.5% of the total unigenes annotated by GO, respectively (Figure 4). The results indicated that most of the sequenced genes were responsible for fundamental biological regulation and metabolism. In addition, 1849 unigenes, which contained cadmium transportation, detoxification and cellular Cd homostasis were annoted based on GO annotation (Table 4). Of these 1849 unigenes, most (1750) were annoted to plant response to Cd ion and 154 were annoted to the Cd transportation which could be related to Cd transmembrane transporter and transporting ATPase activity.

**Table 4:** **GO annotation of unigenes related to Cd responses**.

**GO term name**	**GO term number**	**Total unigenes**
cadmium ion transport	GO:0015691	82
cadmium ion transmembrane transport	GO:0070574	17
cellular cadmium ion homeostasis	GO:0006876	3
cellular response to cadmium ion	GO:0071276	8
detoxification of cadmium ion	GO:0071585	4
response to cadmium ion	GO:0046686	1750
cadmium ion binding	GO:0046870	27
cadmium ion transmembrane transporter activity	GO:0015086	55
cadmium-transporting ATPase activity	GO:0015434	1
Total		1849

**Figure 5:**
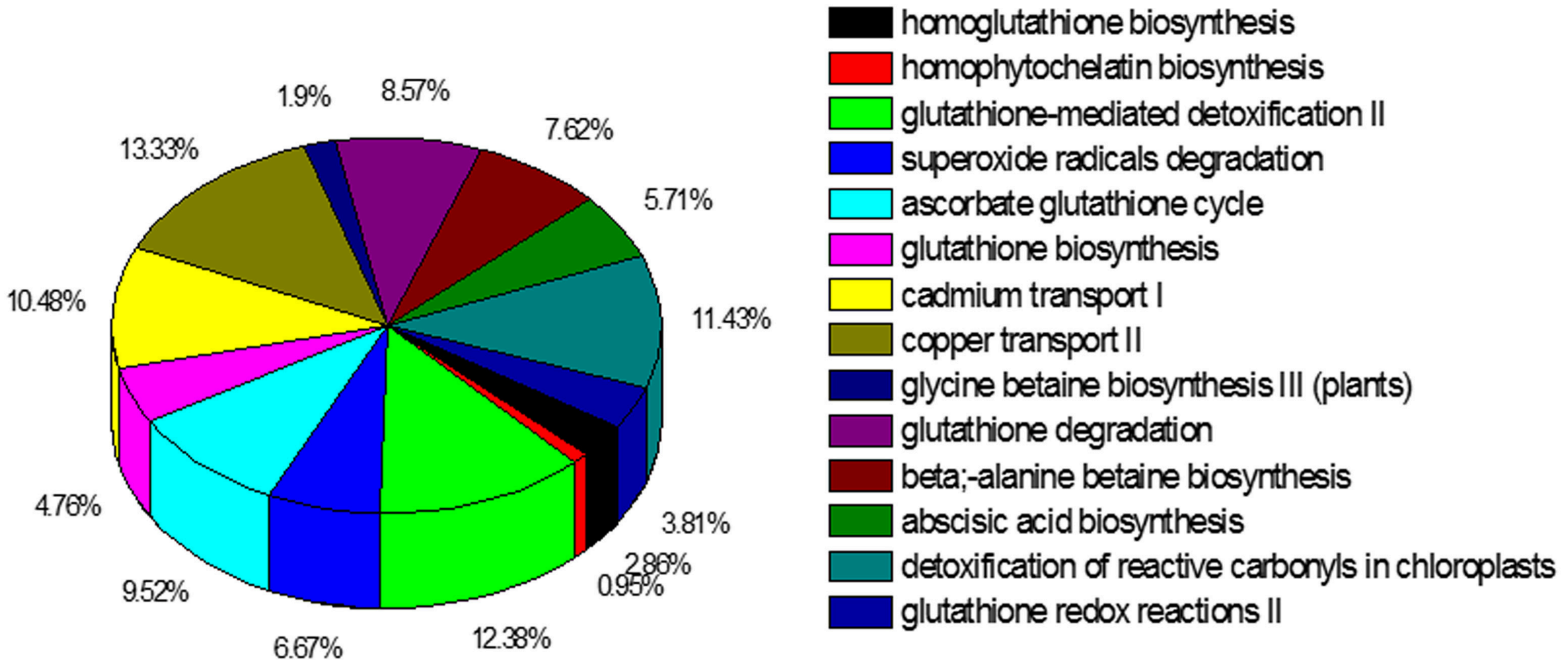
**Cadmium transportation and detoxification pathways represented in the PlantCyc annotation of the unigenes**. In total 13,973 unigenes of the 80,105 unigenes were annotated to 607 pathways.

The KEGG metablic pathway analysis revealed only 10,552 (13.2%) unigenes were assigned to 256 KEGG pathways (File S2). The predicted pathways represented the majority of plant biochemical pathways including metabolism, genetic information processing, cellular processes, and organism systems. The pathways with highest unigene representation were Ribosome (ko03010, 587 unigenes, 5.56%), followed by protein processing in endoplasmic reticulum (ko04141, 382 unigenes, 3.62%) and oxidative phosphorylation (ko00190, 350 unigenes, 3.31%). The PlantCyc database is also a plant-specific metabolic pathway database, which includes experimentally supported, computationally predicted, and hypothetical pathways and enzymes. In total 13,973 unigenes were annotated to 607 pathways using the PlantCyc database, of which we focused on pathways (509 unigenes) (File S3) to identify information on cadmium transport and detoxification in *S. integra* (Figure 5). A major share of the unigenes related to the heavy metal detoxification mechanisms were from the detoxification of reactive oxygen species, including ascorbate glutathione cycle (99 unigenes, 18.10%), superoxide radicals degradation pathway (67 unigenes, 23.34%), and glutathione-mediated detoxification II (41 unigenes, 14.29%). The unigenes related to metal transport pathways were mainly from copper transport II and cadmium transport I pathways. In addition, unigenes from glutathione and homoglutathione biosynthesis, homophytochelatin biosynthesis pathways, which provides the intermediates for the detoxification process, were also annoted according to the PlantCyc datebase.

### SSR marker discovery

In total, 4410 sequences containing 5743 SSRs were identified from 15,007 unigenes (30.8 Mb), with 1026 unigene sequences containing more than one SSR. Most of these SSRs were perfect SSRs, only 353 were presented in compound formation. Mononucleotide were the most common SSR type in *S. integra* genomic sequence representing 42.19% of all SSRs, followed by di- (28.1%) and tri-nucleotides (28.19%) (Table 5). Tetra- and penta- nucleotides were the least frequent repeat types, together representing less than 2% of the total SSRs. The most abundant repeat type was A/T (2329), followed by AG/CT (1049), AAG/CTT (326), AT/AT (277), AGC/CTG (275), and AC/GT (271), respectively. We also found the most abundant repeats in the SSR sequences was 10–20 repeats (1441), accounting for 25.09%, followed by 10 repeats (1197), which accounted for 20.84%. Few SSR sequences were >20 repeats, which only account for 0.66% (38).

**Table 5:** **Frequency of SSRs in *S. integra***.

**Motif length**	**Repeats number**								**Total**	**%**
	**5**	**6**	**7**	**8**	**9**	**10**	**10–20**	**>20**		
1	–	–	–	–	–	1040	1345	0	2423	42.19
2	–	570	386	218	187	157	96	0	1614	28.10
3	911	430	254	24	0	0	0	0	1619	28.19
4	62	15	0	0	0	0	0	0	77	1.34
5	10	0	0	0	0	0	0	0	10	0.17
Total	983	1015	640	242	187	1197	1441	38	5743	
%	17.12	17.67	11.14	4.21	3.26	20.84	25.09	0.66		

### Expression of genes related to cadmium transport and detoxification in different organs of *S. integra*

**Figure 6:**
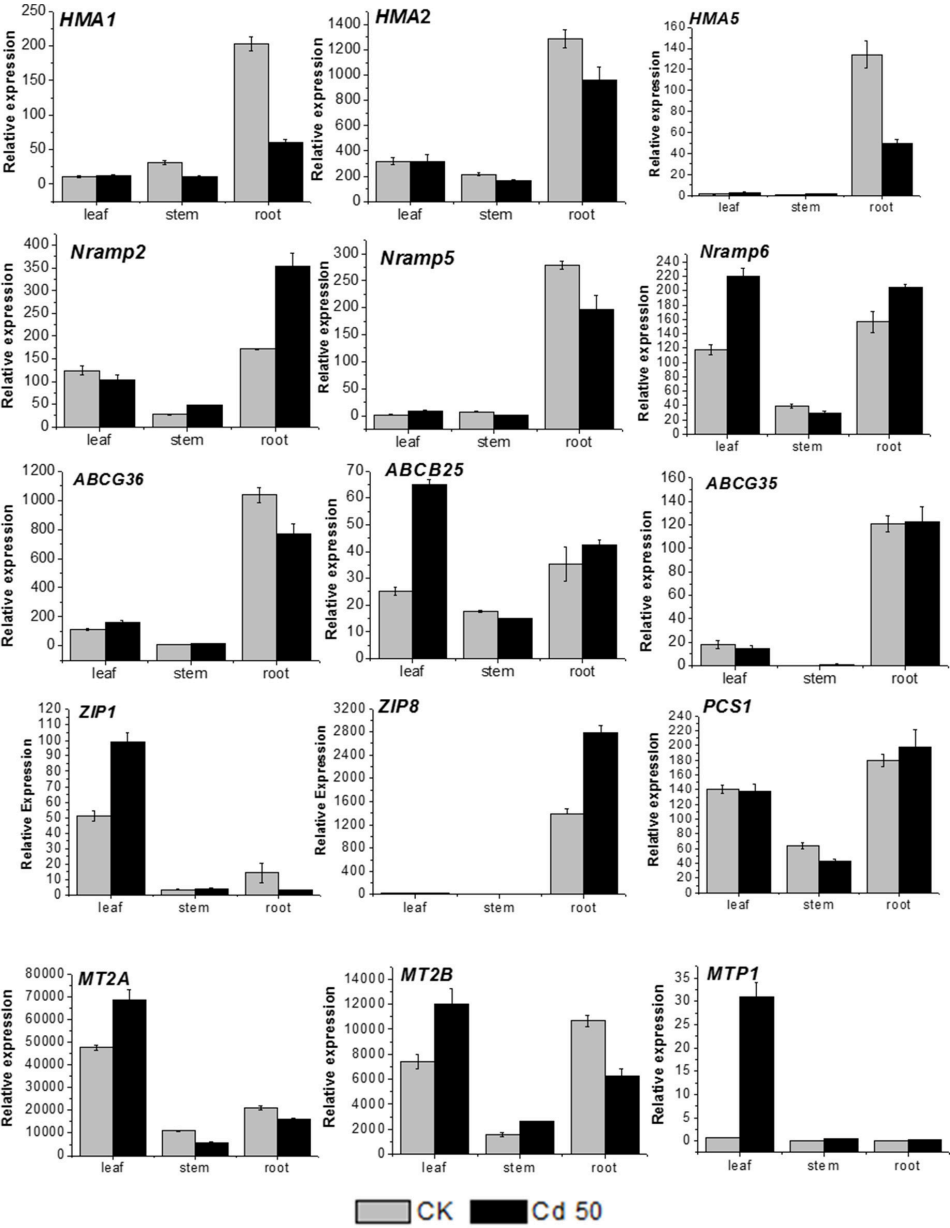
**RT-qPCR analysis of 15 cadmium transportation and detoxification-related candidate unigenes in *S. integra***. The gene names, sequences and the primers used for RT-qPCR analysis are shown in File S1. Standard error of the mean for three repetitions (five biological replicates included) is represented by the error bars.

Several genes related to metal transport and detoxification were selected based on the current RNA-seq results for qRT-PCR analysis to predict their function and expression pattern in different organs of *S. integra*. It is observed (Figure 6) that all the candidates showed various expression levels among different organs under both Cd treatment and the control. In general, the expression level in stems were lower than that in roots and leaves under both Cd treatment and the control. Two unigenes (comp97373_c0, P-type metal ATPase *HMA5* and comp88978_c1, zinc transporter *ZIP8*) were expressed mainly in roots, with very low expression level detected in leaves, and stems, whereas another zinc transportor *ZIP1*(comp107026_c0) and metal tolerance protein *MTP1*(comp690270_c0) were expressed mainly in leaves.

In the roots, three unigenes (comp103862_c0, metal transporter *NRAMP2*, comp63242_c0, *NRAMP6*, and *ZIP8*) were upregulated (*P* < 0.05) by Cd, whereas three P-type metal ATPase *HMA1, HMA2* and *HMA5* (comp93538_c0, comp95545_c0, and comp97373_c0) and metallothionein-like protein *MT2B* (comp62715_c0) were downregulated under Cd treatment. The other candidate unigenes in root showed the similar expression levels between cadmium treatment and the control.

In the leaves, the *MTP1* expression was more induced by Cd, with more than 30-folds higher than that in the control, and five unigenes (*MT2A, MT2B, ABCB25, NRAMP6, ZIP1*) were also found significantly upregulated by Cd (*P* < 0.05). Seven unigenes (*HMA1, HMA2, ABCG36, ABCG35, NRAMP2, NRAMP5*, and *PCS1*) expressed at similar levels under both the normal condition and Cd treatment. Among all the candidate genes, only *HMA5* and *ZIP8* expression were hardly detected whatever under Cd treatment or the normal condition.

## Discussion

Although fast growing woody plants have been widely used for cleaning up HM contaminated soils, limited information is available about the physiological and molecular mechanisms of HM tolerance and detoxification in woody plants (Konlechner et al., 2013; Luo et al., 2016). To date, most of genes involving in HM uptake, transport, sequestration, and detoxification in woody plants were restricted to *Populus* (Marmiroli et al., 2011; Luo et al., 2016). Willow and poplar are closely related species, they are similar in many respects, for example, both species are propagated as clones, and could accumulate moderate contents of HMs in their aerial parts. The complete genome sequencing of *Populus* (Tuskan et al., 2006) has shed light to genomic studies on willow species. However, sequence information of willow is scarce. And what's more, *Salix* have a wider range of genetic diversity than *Populus*, and many of *Salix* are shrubs, which is rare in *Populus*, this may lead to some different response to HMs at molecular level. In this study, we obstained 60,047,711 high-quality reads from *S. integra* on the Illumina HiSeqTM 2000 platform, and 80,105 unigenes were identified by *de novo* assembly. Among these unigenes, 50, 221(62.7%) unigenes were functionally annotated in at least one database of the NCBI Nr, Nt, Swiss-Prot, KEGG, KOG, and COG, and the unmappable unigenes still accounted for 37.3% of all unigenes. Similar unmappable propotion in transcriptome was also reported in some well-studied transcriptomes, for example, 30% in *Triticum turgidum* (Krasileva et al., 2013) and 45.10% in *Dendrocalamus latiflorus* (Zhang et al., 2012) were also not functionally annotated. We speculated these unmappable unigenes might be willow-specific genes or highly divergent genes, or expressed pseudogenes, and noncoding transcribed sequences etc. according to previous studies (Krasileva et al., 2013). In fact, some of these unannotated unigenes, such as noncoding transcribed RNAs, also regulate various cellular processes in *S. integra*, and might have a high chance of corresponding to novel or undescribed genes related to HM detoxification mechanisms in willow.

Based on Nr annotation, the alignments of willow unigenes to *Populus* genome predicted genes revealed 63.96% of annoted unigenes showed significant homology with sequences of *Populus*. While in the recent study on the transcriptome analysis of *S. matsudana*, a Cd-tolerant arbor willow, a higher sequence homology (85.2%) with poplar was reported (Yang J. L. et al., 2015). We proposed this might be due to the genomic variance between shrub willow and arbor willow. A remarkable natural genetic variability among willow species and ecotypes adapted to HMs had been reported in many studies (Laureysens et al., 2004; Puschenreiter et al., 2010). However, molecular marker based classification for heavy metal tolerance has not been documented in *S. integra* population. SSR markers have been successfully used as a methodology in environmental population genetics to detect relationships between stressors and genotypic variability of plant in recent years. Here a large SSRs collection (30.8 Mb) was obtained. These SSRs would provide a valuable functional genomic resource to assess the genotoxicity of Cd in *S. integra*, and to characterize the tolerant and non-tolerant natural populations of shrub willows.

Concerning the adaptation to HMs in plants, woody plants and herbaceous may possess similar physiological and molecular mechanisms underlying HM uptake, transport, sequestration, and detoxification (Luo et al., 2016). However, studies on HM transportation in woody plants revealed they may have a probably more complex mechanism than herbaceous, because woody plants are largely non-hyperaccumulators, and they prefer to accumulate HMs in root cells, instead of transporting HMs to the aerial parts like hyperaccumulators (Migeon et al., 2010; Yamaguchi et al., 2011). Previous study on the HM allocation in *S. integra* also showed Cd prefers to accumulate in roots with more than 3000 mg Cd kg^−1^ dry weight, much more than that in leaves and shoots, and we concluded this preferential accumulation of Cd in roots might be related to the active root morphological development and ion homeostasis under Cd stress (Wang et al., 2013; Yang W. et al., 2015). However, no information on the related genes involved in Cd uptake and transport in roots of *S. integra* was reported by now. In this study, for the first time, we identified many candidate genes related to Cd transport from the *S. integra* transcriptome, including *HMA*s, *NRAMP*s, *ZIP*s, ABC transporters, etc., Although the functions of these genes were well-studied in herbaceous, only few metal transporters involved in HM uptake have been functionally characterized in woody plants (Migeon et al., 2010; Li et al., 2015). It is reported NRAMP family members are important heavy metal transporters that transport metal ions out of the cytoplasm either into extracellular space or into intracellular organelles or storage compartments (Ehrnstorfer et al., 2014; Pottier et al., 2015). Ishimaru et al. (2012) reported *OsNRAMP5* knockdown plants accumulated more Cd in shoots and may also be involved in Cd uptake from xylem. In this study, *NRAMP5* expression in the roots was observed suppressed when exposed to Cd treatment, suggesting *NRAMP5* may promote Cd translocation from roots to shoots in *S. integra*. Whereas, two NRAMP family genes (*NRAMP2* and *NRAMP6*) were significantly up-regulated by Cd in *S. integra* roots, indicating NRAMP family genes may contribute to the accumulation of Cd in the roots of *S. integra*. And interestingly, we unexpectedly found the probably function of *NRAMP2* in *S. integra* unlike previous studies, in which *NRAMP2* was identified as a candidate iron transporter gene only in mammalian (Fleming et al., 1997; Nevo and Nelson, 2006). This result indicated that homologous genes may show differences in gene function, and would provide new insight into the discovery of probably new genes related to HM uptake and transport in *S. integra*.

Although Cd was largely accumulated in roots of *S. integra*, there were still considerable amounts of Cd which could be transported to the leaves and shoots, which made it possible for phytoextraction of Cd by harvesting the aboveground biomass. High accumulation of HMs can cause detrimental effects on plant growth and development (Gallego et al., 2012). While during evolution, plants have developed several strategies to reduce the toxicity of HMs by sequestration and detoxification. Vacuolar sequestration in leaves is a common mechanism for heavy metal tolerance and detoxification in plants (Verbruggen et al., 2009; Ueno et al., 2011). Metallothioneins are involved in metal sequestration in many species (Guo et al., 2003; Turchi et al., 2012). Guo et al. (2003) reported the *MT2A* and *MT2B* in roots of *Arabidopsis* were induced by copper. Hybrid aspen (*P. trichocarpa* × *deltoides*) *PtdMT2B* conferred Cd tolerance to a Cd sensitive yeast strain (Kohler et al., 2004), and the *MT2B* expression levels of *P. tremula* × *tremuloides* correlated with foliar Zn and Cd concentrations on metal contaminated soil (Hassinen et al., 2009). Konlechner et al. (2013) also reported induced metallothionein expression might constitute a general metal response in *S. caprea* leaves allowing higher Zn and Cd uptake. *S. integra* could accumulate 90–288 mg Cd Kg^−1^ dry weight in their leaves, however, molecular mechanisms underlying the high accumulation of Cd in leaves were still uncovered. Here we discovered the expression of two metallothionein-like protein *MT2A* and *MT2B* in leaves were upregulated when exposed to Cd treatment, indicating metallothionein-like protein might be involved in the leaf sequestion of Cd in *S. integra*. Cation-diffusion facilitator genes (also known as MTPs) are also important for vacuolar sequestration (Verbruggen et al., 2009). Blaudez et al. (2003) reported *MTP1* from hybrid poplar (*Populus trichocarpa* × *Populus deltoides*) was characterized and functioned as genes related to Zn tolerance. In our study, there is a nearly 30-fold increase in *MTP1* expression in the leaves of *S. integra* when exposed to 50 μM Cd (Figure 6), indicating *MTP1* may play a key role in the accumulation of Cd in the leaves of *S. integra*.

We also examined two ZIP family genes (*ZIP1* and *ZIP8*) and found the basal expression level of these two transporters were quite different in different organs. Members of the ZIP gene family are capable of transporting a variety of cations, including Cd, iron, manganese and zinc (Guerinot, 2000), but very few members were characterized and functioned in *Salix*. Konlechner et al. (2013) detected overexpression of *ZIP6* in leaves of *S. caprea* by Zn and Cd, suggesting ZIP family gene play an important role in Zn/Cd transportation in *Salix*. In this study, we identified *ZIP1* and *ZIP8*, which might be involved in the Cd transport respectively in the leaves and the roots of *S. integra*.

## Conclusions

We have generated a large collection of annotated unigenes from the Cd accumulator *S. integra* transcriptome, which should provide more opportunities for studying heavy metal tolerance and detoxification in *Salix. S. integra* could accumulate a large amounts of Cd mainly in their roots. *NRAMP2, NRAMP6 and ZIP8* would be involved in the Cd uptake and transport in the roots, thus contributed to the high accumulation of Cd in root cells. *MT2A, MT2B, MTP1, ZIP1*, and *ABCB25* might play a key role in the Cd sequestion and detoxification in the leaves of *S. integra*. Besides the similar function of these genes to rice or *Arabidopsis*, or *Populus*, the present study provides a basic understanding of some specific expressed genes in *S. integra*, suggesting their special roles during HM uptake and detoxification, this would provide novel insight into the new candidate genes discovery in willows. However, our results on the expression profiles of candidate genes are largely based on RT-qPCR, which is limited to the comprehensive understanding of the differentially expressed genes when exposed to Cd stress. Although we determined the tissue-specific expression of selected genes, we still could not know the exact location of these genes. Therefore, further experiments, such as DEGs sequencing and subcellular localization of these genes should be carried out to demonstrate the function characterization of HM related genes in *S. integra*.

## Author contributions

Conceived and designed the experiments: SW, XS. Performed the experiments: SW XS. Analyzed the data: SW, HS. Contributed reagents/materials/analysis tools: YC, HP. Wrote the paper: SW.

## Funding

This work was supported by grants from the National Natural Science Foundation (NSF) of China (31400526), Social Development Program of Zhejiang Science and Technology Hall (2016C33043) and Central Public-interest Scientific Institution Basal Research Fund (CAFYBB2014QB035).

### Conflict of interest statement

The authors declare that the research was conducted in the absence of any commercial or financial relationships that could be construed as a potential conflict of interest.

## Supplementary material

File S1 **Gene names, sequences and primers for RT-qPCR**.

File S2 **Pathway assignment based on KEGG**.

File S3 **Pathways and unigenes related to metal transport and cellular detoxification based on PlantCyc database**.
