# Supplementary material for: Transcriptome Sequencing and Expression Analysis of Cadmium (Cd) Transport and Detoxification Related Genes in Cd-Accumulating Salix integra
Source: Front Plant Sci. 2016 Oct 28;7:1577. doi: 10.3389/fpls.2016.01577 (PMC5083712; doi:10.3389/fpls.2016.01577)
Supplement: File S1 — Gene names, sequences and primers for RT-qPCR. [file DataSheet1.DOC]

1. Unigene 75580_c0

Metallothionein-like protein (MT2a )

5’-TTTTTTTTCCTCAAGAAATCGCCTCAATTATTATATCATTTAATAGCGCCCAGTTACAACCCACCAGTAATTTATTTTCTTGTATAGCTGTAATAACAATCTGAAGTGATGATAGGATGAATGGGCATGCGAATCCCCCCATACATGCACATACAAACACAGACAGCATGTAAATATCACAGTAACACTACCATTTTAATTATACAAGAAAGAGAAACGAACGACAAAGCAGCTGGCTCGACTTTAACCAGAGAGGAATCCCCAGCAGCTGATAATCCCTGGAAGATTTGCAGAAAACAAGCCACGACTGCTAGCTACTTTATTGAAACTTTTCAAGAACAAGTGACCACCCTTATAAACAAACACCTGGTAGACTACATATTATTTCTTGAATCAACGCAGCCAGCGGATCGATCTCATTTGCAAGTGCAAGGATCGCAGGTGCAGTTGTCTCCACACTTGCAGCCATTCTCGGCTCCCATGACCATCTCAGAAGCCCCCTCAAAGTGATTCTTCTCCGGTGCGACACCAAGAACCAGAGTTTCTGTGGTGATCGTCTCGGCGGAGCTCAAGTCAGGGTACATCTTGCACCCTCCACAGCCGCTGCCGCACTTGCAGGCAGAGCCACACCCACAGTTTCCTCCACAGCAAGACATCTTCACGAAGATCAAATTAAGAGTTAAGGAGTATTTAGCTGAATGACACTTGTGCTTGAACC -3’

MT2a-F 5’-TCCACACTTGCAGCCATTCT -3’

MT2a-R 5’-CTGTGGAGGGTGCAAGATGT -3’

2. Unigene 62715_c0

Metallothionein-like protein (MT2b )

5’-GTCTTGCTGTGGAGGAAACTGTGGCTGCGGCTCTGGCTGCAAGTGCGGCAGTGGCTGCAACGGATGTAGCAGCATGTACCCAGACCTGAGTTTCTCCGAGACCACCACAAGCTCGGCGAGCATTGCCGGTGTCGCTCCGGTTAGGATGTTCTATGAGAGCTCGAAGATGAACTTCGGTGCGGAGAATGACTGCAAATGTGGATCAAACTGCTCCTGTGATCCATGCTCCTGCAAATGAGAAAACGTTGCAGCATGGCTCCAGCCAAGCAGTTTTATGGGACTATAATAAATAAAAAGAAGTCTGGTCGCACCATGTTTGTCTAATATAGTACTGTTTGCTGCATATTAAGTACAGTTAGCTAGCCATGGCTTCCTCAAATCCTTTCTATAGGATTTCATTCGATGGCTGCTAATCTGTACGTGTCTTGTGTTTCCTGTTGCTTTGCTGGTTCCATGGAGCTCTTCTATATCATTAATTGGAAGTTTGAATCTGTCGTGCGCGCGTTGACTGAAATGCTCCTAATTAATGCTTGATTCTGCTTTTATTTTTAGTAAAAAA-3’

MT2b-F 5’- GTTTCTCCGAGACCACCACA -3’

MT2b-R 5’- AGTCATTCTCCGCACCGAAG -3’

3. Unigene 690270_c0

Metal tolerance protein 1( MTP1)

5’-TTGACTTGTCTTGAGACTTTGAGTGATCGACTCGCTAGTTTCAACTAGTTCCTCGTCTTCATCATCATCAACGTCGCTGTCGTCGTGTATGTGCACCAAGTGACTGTGATTGGCGATGTCGTGCTCGTGATCGTGCGCGTGTCCATGGCCATGGCTGTGGCCAGCGTGACTATGGAAATGGCCGTGTCCATGGCCGTCAACATTGAGCGCTATGGCCATGATTATGTTAACCAATAGGCCAGCCGTGGCGGTGATTACCATAACCAAGCTGTCAATCTCGAACTCGCCCGTTCGCAGACGCTCGACGGCCATATAAACGAGCAGTCCGGTGACGAGCCAAATCATGAGCACCGACAAGAGG-3’

MTP1-F 5’- CATCATCAACGTCGCTGTCG -3’

MTP1-R 5’- ATGGACACGGCCATTTCCAT -3’

4. Unigene 93538_c0

heavy metal ATPase (HMA1)

5’-GAAAGCTGAGAAAGTGAAGGAATTACAGGCTACAGGCTTTACCGTGGCCATGGTAGGTGATGGTGTAAATGATTCGCCAGCTCTTGTAGCAGCCGATGTTGGGATAGCAATCGGTGCAGGCACAGATATTGCTATAGAGGCAGCTGACATAGTTCTGATGAGGAGCAGTTTGGAGGATGTGATTACTGCTATAGACCTGTCTAGGAAGACCTTTTCCCGGATTCGCCTAAACTACATATGGGCTTTGGGATATAATATCATCGGCATCCCAGTAGCTGCCGGGGTGCTTTTTCCTGGCACTGGACTCCGTTTACCACCGTGGGCTGCTGGAGCTGCAATGGCAGCATCTTCAGTCAGTGTGGTTCTGTGCTCCCTCTTATTGAAGAATTACAGAAGGCCCGAGAAACTGGAAAATCTAAATATTCACGGGATAAAGATCGAGTGATTAATAACACTGGTTTCGACTCAGGTTTTCTGAATAGATGTTCATCAGGAGTTGATTGAGAGCAATGTAGAC-3’

HMA1-F 5’- GCACTGGACTCCGTTTACCA -3’

HMA1-R 5’- CAGTTTCTCGGGCCTTCTGT -3’

5. Unigene 95545_c0

Cadmium/zinc-transporting ATPase (HMA2)

5’-CAGATGGATTGACACCATATACAAGGCCTTGACCCTTGATGGATTGAAAGGAGAAGTACCAAATAACAAAAATCACTGCTACAAAATGTTAAACAGAGGATTAAGGTTGTCATCATTCAGATTCATGGCTAATTATTAATGTACAATAGTTTTAATCGATACAGAATGATGAAATCACAAGTTATGATGACATAAAATATTTCTGTTAATATCGAGCACTTCTTAATTTCATTTCATTCATTAATTAATTCTGGTTCTTGGTTACTGAACGTGTTTCACCAAAATTTCCTAGTTCCTTCGCTAGAACCAAAACAATATTCGTCTAAAGATCTTCACACCTGTTTGATTTCTGAGAACCCTCCACCAAAGCCAGTGGCGAAGTGTCCGTGTCCATGCTTACTGCAACATTCTTTCATGTAGCTCTTGCAGCATCCACCAGTGTCTCTCTTCTCCAAGCTCAAACAAGCATGCATGGTTGTTGGCTCAATATCAATAACAGTGTGAAGTGGAGCGCAGCCTGTACAGCACTTCTTCAACTCTTGGTGTACCTCTTGGTGTGGATGCTGGGGACTGCAACTGGATTCAACAATCTCTCCTACTGACTCTTCATTCTTGGGAATGTCAATGGTGTTCTTGCAGCTTTTCTTGGAGCAGCAATTGTCTGTCATCCTTTTACCGCCCTCTAAATTTTGAGAAGAATGACTTGAATGGTGATGAATACCAAGATGAGCAGCAGCAGGTTCACTGTGTGAATGGCTGCGTGGATGATTTGCCTCTGTGTCAGGATTGACTGTGTTACAGCAACCATGATCATGGTGCTTGGCTTCGTGAACTCCACTAGCATTAGTTGCTACACATACATCATGGCTCTTAGCTGAGTCAGCGCACTCTTGGTTTCTGCATGAGCTGACACTATGTTGGCTAGTCTGGCATCCTGAGGCAAGGCTTGAACTTTGAGCCACACAATGCTCCTCCACAACTTTCTGGCTAGAGCAACATCCTGTGTCGCAGCTTGGATTATGGGCCACAAGCTTCACCTTCTCAACTTTCTGGCTAGAGCAACATGCAGTGGCACAGTTTGAATTAGGGGCCGCAAGCTGCACTTTCTCAACTTTCTGGCTAGAACAACATCCTGAGGCACAGTTTGAGTTTTCAGCTGCAAGCTGCACCTTCTCAACTTGTGGACTAGAGCAACACCCTGAGGCACAGTTTGGATTTTGAGCCCCGCTCTGCACCTGCTCAACTTTTGGGCTGGAACACTTTCGAGCCCCACATTGCACCTTCTTGACTGTTTGGCTAGAGCAACAATTATGTTGATTGTGTGAAGAATTCTTATTACCATGTTTGTGCGAATGCGATGCACCTGATGATTTGCTGCATTTTCCTGGATGATTGTGTGCGCCTCGTAAAAGCAGCATGCTGTTCAAAATGACCAGCAAGCATGTACCTACATCAGCAAGAACAGCTGCCCAAACAAGTGGGTGACCAGCAACGGCCAAGGCGAGGATAGCAGACTTTGTAGTCATGGACAAAATCACATTCTCAATCACTTTTCTATGGGCCTTACGGCCAAGCCGGATGGCTTTTGGTACTTTCCGGAGATCATTGGACATAAGAATTACATGTCCTGTCTCTGTTGCAAGTGCTGAACCTGAAATTCCCATTGAAATGCCAATATCAGCAGTAGCTAATGCAGGGGCATCATTCACACCATCTCCAATCATTGCTGTTGGTCCTTCCTTCTTAAGCTCTTTAATAATAGCCGCCTTGTCTTCAGGAAGGAGTTCTGCATGAATAACGTCAAGAGCATGCTCTAGCTGTTCATGTGCATACATGGCTGCTGCTTCACTATCTCCTGTAAGCATTGCAGTTTTAATGCCTAGTGACTTCAGTTCCTTAATGGCCTCTGCAACCCCGGTCCTACAGCTATCAGAGAGACCAAAAATCCCTGCAAGAGTTGTTCCATAGTACACATATCCGACAGACTTTCCTGTCTTCTTGTCACCTTCTGTTGTTGGAACTGTTCCAGATGCTCTGTGAGCAATCTTTTTGTTCCCTATATATATATCTTTCCCTTCAATTTTTCCTTGAATTCCTTCTCCAGGAAAATTTTGAAACTCCTCCACGTTCTCAGGCTGTGGCTCTATTGAATGCATCCTTCCATAGTCAACAAGTGCAGCCGCCATAGGATGACTTGATTTGCTCTCGATGCTTGATACCCAATAGAGCAATGTGTCAAAGCTGATATCATTGCAGAGAGGTTGGAAATCTGTGACAGCAAATTCACCTCTTGTTATGGTACCGGTTTTGTCGAAAGCCATGGCCTTAATCTTGCCTAGTGTTTCAAGATAGTCTCCCCCTTTAATCAGAAGACCAGCTGATGCCGCCTTTGTAAGAGCACAAAAAGTTGCAACTGGTGTAGACAGGATTAGAGCACATGGACATGCACTGACTAAAACAACCAATGCTAAACGAAACCAGCGATTACGGTCATGAAGTCGTAGTGCAAGCGGTATCACTGCTAAGCTGGCTGATATAATAATCACCACTGGAGTATAGTATTGAGCAAATTTGTCTATGAACCTTTGAGTTTTAGACTTGCTGTTTTGAGCTTCTTCCACAAGCTTTGCCATTTTAGCCACCACACAGTCTTCAGCTAGAGCAGTAGTTCTGACATTCATATAACCATTCAGGTTAATGGTGCCTGCCCAGACAGTTGAATCCACCTGTTTGGGGACTGGAAATGACTCTCCAGTTAACGTTCTCTCGTCCACTTCACAGTTACCATCGACAACAACTCCATCAATGGGAATAATTTCACCAGCCTTAACTGCAAGAACAGTGTTTAACTTGACCTCGTCAGCATCCACTTCTTCCCCAGTTTCAGCTATGACTGCTTTCTGAGGAGCTATGCTCATCAATGATGACATGACAGCATTGGCCTTGTGGCTTGCCCTTGACTCTAGCCATTCTGCAATTGTGAACAGGAAGACAATGGTTCCTGCTTCAGTATAATCATTCATCGCAATTGTTCCTATGACTGCAATAAGCATAAGAACGTTGGTATCAAGCCTAAAATGCCTCAAGGAAGCAACAGATTTCAAGCAGATGGGAAGGATGCCAACAGCCACTGCTCCAATAGCAAACCAACGCAAAGGATGATAGACATACTTCAAAAGTGATAGCAAAAGTAACACACCACAAACCACTACATATGGGCTAGGCCATTTCTTTTGGTGCTTGGTCTCTCCATATGCTCTAATATTTGCTTCCAGTCTTGCTTGATTTAGAGCCTTAACAATTTGAAGCTGAGAAATTAGGAGATCGTCATGGATAACAATCACTGTTCTTGTTGGGATAATCACTGAGTACTCCTTAACACCATCAAGCGACTTGAGGATATTCTCGATCAAAGGAACCTCTGAAGAACAGCAAAGGCCTAGCACATCAAAGTAGCTTTTCTTCAACTTTTTGGTAGCTTCCGTGTTGCTTTTTTCCAACTGGGCAGCCATTGTTTTTATCTCCAAATCTCTCTTGAATATGAACGAAGGATTTGAGGAAGATGAAGCTTTCTTTTGAGGAGGAGAGAAGGAGCGGGTAAGGAGATTTATAAGCTGAGAAGATTGGAGTGGAAG-3’

HMA2-F 5’- TGCGTGGATGATTTGCCTCT -3’

HMA2-R 5’- AGAAACCAAGAGTGCGCTGA -3’

6. Unigene 97373_c0

Probable copper-transporting ATPase (HMA5)

5’-TTTTTTTTGCCTTCAATTTGATACTTGCATTGACATTTAAGAAGAATCAACAGTGCATTTTGCCACTAGAAAATGTATTTATACATAAAATACCTAACAAATATCTACAACTTTATTGTACAGACTATCTTCACTTCTAAAGGAGCACACGCTTAAGTTTATCACAAGGCAAATTCCACTCTTACACTAATTTTATACAGAAAACTGAATGGAAACTGTTGTGAATTGATCACTCAATCCTTATTCCACCGATATATAGATTTTCCAGCTTCTTGGGCCTTTTATAATTCTTCAACAAGAGTGAGCACACAACAACACTGACTGAAGAGGCTGCCATTGCCGCTCCAGCAATCCAGGGTGGTAAGCGAAATCCAGTGCCTGGAAAAAGGGCCCCGGCAGCTATTGGAATCCCCATGAGGTTATAGCCCAAAGCCCAGATATAGTTCAGGCGGATCCGGGAGAAGGTTTTCCGGGAGAGATCTATGGCAGTAATCACATCCTCCAGGTTACTCTTCATTAGAACTATATCAGCTGCCTCTATAGCAATATCCGTGCCTGCACCAATAGCCATACCAACATCTGCGGCTACGAGTGCTGGTGAATCATTTATACCATCACCTACCATTGCCACTGTATAGCCTGCAGCCTATGAATGAAAAAAATTTGAAAGTCCAGAAACTAATAAATTGATAGAGAACATGAAAAAACAAAGTTCAATTATGCACCTGCAATTCCTTCACTTTCTCAGCTTTCTGCACAGGCTTAGCTTCTGCGATGACAGTTTCAATCCCAACTTCCCTGGCAATGGAATTGGCAGTTCCCCAGTTGTCACCTGTGACAATGATGCTCTTAACTTTCATGGACTTGAGAATGGAAATGACTTCACGAGCACCCGGTTTCAATGGATCTGATATTGCTAGAACTCCAGTGACTTCCCTGTCAATAGATACTAAAATCCCAGTTTGAGCCATCCCTTCTGTTTCTGCGAGGATCTCTTCTGCGTCAATTGAAATGGGGATGTTGTTTTCCAACATCAAGCTTTTATTTCCCATAATCACTTCCTTGTTTCTGACGATAGCCTTCACTCCGTGGCCGGTAATGGACTCAAAATCTTGAGCTTCTGGCCACTTAGGGTTCTCTTCATCTTCTCTGAATTTCTTGGCATACTCTACAATGGCCTTGGCCAATGGGTGCTCACTGTTTACCTCAGCTGCAGCTGCGAGCTCATAAAAATCCCTCAGTGCCAAATTTTTCAAGAGCCTTGTGCTAACAACCACAGGCTTTCCAATCGTAAGAGTTCCTGTCTTGTCAAAGACAACGCAATTCACCTTGTGTGCACTTTCTAATGCTTGGCCACCTTTGATTAGAACACCTTGAGATGCACCTACTCCAGTACCAACCATGACAGCAGTTGGAGTTGCTAATCCAAGAGCGCAAGGGCAGGCTATGACCATAACAGAGATTCCGAATTGGAGAGCAAGCTGAAAGCTATCCATGGACTTCGGTATCCAAGAGCCTGGATATCCATGGAACTTTCCAGCTAGAAACCACGCGAGCCAGGTTGAAAAGGAAAGAATAATAACAAGAGGAACAAAGTATTTGGAGATACGATCAGCAAACTTCTGGACAGGAGCTTTAGCCATCTGAGCTGACTCAACCAGTCGAACAATCTGTGAAAGAGCACTCTCCGATCCAACCCTCGTCGCCTTAATATGCAGCACCCCATTCTCATTCACAGTCCCTCCGATCACTGTGTCACCTTTCCTCTTTGCAACTGGCCGTGCTTCTCCTGTTATCATGCTCTCATTCACGTGACTCTGCCCCCAACTTACAAAACCATCTGAAGCCACTTTTGCACCAGGTATGACTTTAATCAAATCATTCCTTTGTATCAACCGACTATCGATTTCTTCTTCGCTGATAACATTTCCTTGATCATCCAAAGCCAGCAATATTGCAGTGCCAGGTGCCAAGTCCATAAGCTTGGCAATGGCTTCGGATGTCTTTCCCTTAGCCAGAACCTCGAGATACTTCCCGAGAAGAATGAACGAAATAAGCATTGAGCTGGTCTCGAAGAAATCCGTGGACTCAAAACTTGGGGAGGTAGCAGATCTCAACACCAAATAGACTGAATAAAAGTAGGCTGCATTTGTTCCTAAAGCGATCAACACATCCATGTTAGGAGAGCCGTTCCTCAGAGCTTTATAAGAACCTGTATAAAATCGACGGCCTATAATGAACTGCACTGGAGTAGATAACACCCACCTCAAGATTGCTCCTATAGACAGCATATTGACTATTTTGGTGTCTAATGCATGCTTAATTCCAGGAATGTACATGAAAATCATGGATATTAAAAACACTGGAACTGTGAAAACCAAACTCCATAGGAAAGATCGGCGATATTGTTTAATCTCCTCATGTCTATGGCTTTCTCTTCCTCCTCCTTCAGGAAATATCGTTGCCTTGAATCGCCCTGACGTCCCAGTTGATTCGATCACATTGATAAAATTTCTAGGACCAGTTACATCTGGTTTGTAAGACAGGGAAATTTTGTTGACTTCAGGATCTATGTCTATACTTTGAACACCCGGGAGAGCCTGAAGAGAATTTTCAATTATTCTCATTGAATTCTGTGTTCTTACTCCATCAATTTTGAGCCCTATCTTTCCCATGTCTTCCCCAGTACTAAGCAGTATGGCCTCGAATCCAGTTTCGTTTATTGCTTCCAATATCTGATTGTAGCTCAGGATATTTGGATCATAATGAACTTCTGCTTCTTCAGTTGCTAAGGCTATTTGAGCCTTCTGCACACCTGAAATTGCTTGCAAAGCTTGTTCAACAGTGGAGGAGCAGGAAGTGCAGGTCATTCCATTAATACGAACTCGGCATACTTGAGTGGATCTATCACTGGTCCCTTCTTCTTGAATCAATGTAGCCTCAAATCCAGCATCTTCAATCGTCTCCCGAATGGTCTGTTAACGAAACTGGGGTAGAACAGAACTTGAGCCTTGTTGTTCAAGACATCAACAATAGCCTCTTGAATCCCAGGAAGCCTCTTGACAGCTTTCTCGACAGATCCAGCACAAGCAGAGCATGTCATTCCCATCACAGAAAACACTGCCTTCGCCTCTGATCCTTCCACGTTCGTTTCTCCAGCCGAAACCCCTCTCGGGTACTTCGGCATCGAAGGATACCTTGGCCTCGGTGATAAATCTCCATAGCTTCTCTCTTTGCGAATGCATGCTAATGCCAAGAATTTGGTTGCCATGAAATCAGTGTTTAGGTATACGTTATCGTCTTCCAAACTACCTGCAGCACAGGTAGATATAATTG-3’

HMA5-F 5’- TCACAG TCCCTCCGATCACT -3’

HMA5-R 5’- CCTGGTGCAAAAGTGGCTTC -3’

7. Unigene 88978_c1

Zinc transporter (ZIP8)

5’-CAGGAGGGAGTCACATTTTCTTTCTGTATAAATGAAAACACGATAGCAATATTTTCACACAGAATCTGCCTGCCTTCATCTTCAATCCCTTTTCCTTGATCACTTTGCAAACACATTTTCTCTTGATAGCATCACCATGCAGAGTTCTAGCAGATTTTACTTCGAGCTCTTCTGCTTGCTCCTGCTACTCCCTACTCTTGCTTTAGGAGAATGCACATGCGATGCAGGAGGAGAAGAAGACACAAATAAATCCGAGGCCTTGAAATACAAAGCCATAGCAATTGCTTCGATCCTTTTTGCGGGTGCAGTTGGAGTTTGTCTTCCAGTTCTTGGAAAATCGATCCCTGTTTTAAGCCCTGAAAGGAGTGTTTTCTTCATCATCAAGGCTTTTGCGGCCGGTGTCATATTGTCGACAGCCTTTATTCATGTGCTTCCTGATGCTTTTGATAGCCTGACGTCGCCATGCCTTGGTGAAAATCCTTGGGGTCAATTTCCCTTCACGGGTTTTGTGGCAATGATGTCGGCGATTGGGACTTTAATGGTGGATTGTCTTGCCAGTTCTTACTTTACACGGTTGCACCTCACCAAGGCTCAACCAGATCAGAGTGGGGACGAGGAGAAGGCAGCAGGAGAGGCTCATGTTCATACTCATGCAACTCATGGCCATTCTCATGGCATCGTGGATAGCTCTGCTGCTGCTCCATCTCAGCTTATTCGCCAACGGGTTATTACTCAGGTTTTGGAGTTGGGAATTGTGGTGCACTCTGTGATTATAGGAGTTTCTGTAGGAGCTTCTGCAAGTCCCAAGACAATAAAACCTCTGGTGGCTGCCCTGAGCTTTCACCAATTTTTTGAGGGTATAGGACTCGGTGGATGCATTACTCAGGCAAAATTCAAGACCAAAACGATAGTGACAATGGGACTCTTCTTCTCTCTAACAACACCGGTTGGAATCGCAGTGGGCTTAGGCATATCAAACGTGTATAATGAGAGCAGTCCCAACGCTCTGATTGTTGAAGGAATTTTTAATGCCGCATCAGCTGGTATTCTAATCTACATGGCTCTTGTGGATCTTCTGGCAGCTGATTTTATGCATCCAAGAGTGCAGAGCAATGGAGCTCTTCAGCTTGGGGTCAACGTTTCTCTTCTTCTAGGAGTTGGCTGTATGTCTCTCCTTGCCAAATGGGCTTGATCTGCAGTATAGCCCATCCTTTTACTCGTGTTTTTTCTTCAATTTTTTCTGACTTGGCTTCGGCTTTGGCTTTGGCTTGGTGAAGATAGAGAAACTTCTTGGTTTTGTAAATGTT-3’

ZIP8-F 5’- GGCCGG TGTCATATTGTCGA -3’

ZIP8-R 5’- AAAGTCCCAATCGCCGACAT -3’

8. Unigene 79269_c0

Zinc transporter (ZIP1)

5’-TCCGGTTGCTAGGATAACACCAGCAGCAAATGCCTTGATCATAAAGAAAATATCATTTTCAGGCCTAAAGGCTTTAATTTTCTTTCCTAACAAGGGGAGGCTAACGCCAATAGCACCAGCAACTAGAATTGAGAGTATTGAGCCTAGTTTAAACTTTAGTGCTTCACCTTTGCCATGTTCTAAATCTTCAACCTCACACGTACACTCACAGGTGACCATAGCAGGGTAATGAAGAAGAATCAAAAG-3’

ZIP1-F 5’- TAACAAGGGGAG GCTAACGC -3’

ZIP1-R 5’- ACCCTGCTATGGTCACCTGT -3’

9. comp63242_c0

Metal transporter (NRAMP6)

5’-ACTCTGATGCAGATGATGATGCAGTAAGAACCTGTTTGTTTTTGTCTTGCTACTCTAATAAAGTAACTGTTTTTTCATGTTGAAACTTAGATACATTACTTGCTATCTTAAACGCAGTGATTGTGGAGCGATTCAATATTGTTTGCTGTTAACAAGTTTTTGCTCAAGTTTGTAAACTCTTTATATATGCCTGATAAGTCACTAATTGTCATGAAAGGTTTGTTAGTTTGTTGGTGCTAAGGTTTGTTCATATTATTGATGTGCTGCTTTGTCTATGAAATCAACGAATTAGAACTAGCAAATCATCATTTAGCATCATCAAGAGGCTGCGTGGAAATTGTTAATTGAACTTTGTTTTTCTCTAGCTATTTAAACGGTTTGTTTTTTTAGCAGCAGTCCATTAAAATGTGAAAATTTCCTTTCTGATGTATTTTTCCGTGCCACTCCTTTGGATGAAGGCCGCGCCCTCATCAGCAGGCAG-3’

NRAMP6-F 5’- TTGCCAGACTCGTTG CAG AT -3’

NRAMP6-R 5’- TCCATGGCCATCGAAAGCTT -3’

10. Unigene 103862_c0

Metal transporter (NRAMP2)

5’-CTTACCATCGTTAAAAAAGACACAACCAACAACCAGGACTCCATAAAAACCATCTATCCAATCCAAACATCCTCACTAATTCACATCGGACCTCGCCATATCGGAGATTAAGGTCACCCCTGTCCACTCCACTCCACAACGGAATATTCTAATTTAAACGGAATTTCATTAAATGAGTTCGCCATCTGGAGTAGAAGATTCCAAAGAAGACGAAAAAGACGAGGAATCGAACCGTCTGTTACCGTTACCATCCTCAGCTCAATCTCAATCTCAATCGTTAAGAAGTGAGGAGGATTCCGATGAAGTAGCATTCGAAGCGCGAGAGAAAATCTTGATCGTTGACGTGGAAGAGCCAGACTCAATTGACGCCGTTGATTATGTCCCGCCATTCTCGTGGAGAAAACTCTGGTTATTCACAGGACCTGGGTTCTTAATGAGCATAGCGTTTTTAGATCCAGGGAATCTAGAAGGAGATCTTCAGGCAGGAGCGATTGCAGGGTACTCGTTGTTATGGCTCTTAATGTGGGCAACGCTCATGGGGTTGTTGATCCAGATGTTGTCAGCTCGTGTCGGGGTTGCGACAGGGCGGCACTTGGCGGAGTTGTGTAGGGACGAGTATTCGAATTGGGCCAGGTATGTTTTGTGGTTTATGGCGGAGGTGGCACTTATTGGTGCTGATATACAAGAGGTTATTGGGAGTGCTATTGCTATTCAGATTTTGAGTAATGGGATCTTGCCGCTTTGGGCTGGAGTTCTAATTACGGCATCGGATTGGTATGAGCTGCATTGAATTGATCGTGGTTTAAAATTAACTGGGTTTCGCTTGATTTACTCTTGAATCCAAATTCTATTGTTTTATTCTCCATGTTTTTTTAGGATCTTGACAGGTTTTATTGAAGCTGGCTTGAAATAAACTGAAAATTTCGGATTGAGTTATATATCTTCGCTGTGAAGTTGGGCAATAAGTCAACATTGATAGCTAATTGTCACTTGTATCTTTTGGGATAATCCTCTGATGCTTTTCTATTTTAGTTCTGTAAGATAGTAGTTAATATTTGCACTTGCTTGATGATTTGTTTTATTAACTAGGGTTTTTTTGTTCTCTTTTTTGTGTGGACAGTTTCATGTTTTTATTTCTAGAGAATTATGGAGTAAGGAAGTTAGAAGGTGTTTTTGCGGTTCTGATCGCGACAATGGCTTTATCATTTGCCTGGATGTTTGGTGACACCAAGCCGAGTGGAAAAGAACTTTTAAAGGGTATATTAATTCCAAGACTTGGTTCAAAGACAATTCGGCAAGCTGTGGGTGTGGTGGGTTGTGTCATAATGCCGCACAATGTGTTCTTACACTCTGCTTTGGTACTGTCCAGAAAGATCGATCCTCAGAAAAAAGCCCGGGTTCAAGAGGCGCTGACTTACTATTCAATTGAGTCCTCTGTTGCTCTTTTTGTCACTTTTATGATTAATTTGTTCGTTACAACCGTGTTTGCTAAGGGGTTTTATGGTACTCCACAAGCCAATAGTATAGGACTCGTTAATGCGGGGCAGTATCTCGAGGAGAAGTACGGTGGAGGACTCTTTCCAATTCTTTATATCTGGGGTATTGGCTTGTTGGCTGCTGGACAAAGTAGTACAATAACTGGGACCTATGCTGGGCAATTTATCATGGGAGGTTTTCTCAACCTGCGTTTAAAGAAATGGATGAGGGCATTGATAACACGGGGTTTTGCTATTATCCCAACTATTATTGTTGCTATCATTTTCAACACATCTGAAGCTTCATTGGATATTCTGAACGAGTGGCTTAATGTGCTCCAGTCGATGCAGATTCCTTTCGCACTTATCCCTCTTTTGACCCTGGTAGCCAAGGAGCAGATCATGGGGGTATTTAAAATCGGGCCTGTTCTTGAGAGACTGGCGTGGACTGTAGCGGTCCTGGTTATCCTGATCAATGGGTATCTTTTGATTGATTTCCTTAAATCTGAAGTTAAGGGCTTGCTCTTTGGCTTTCTGATTGGAAGTGGCGCGGTTGCATATGTATCATTTATCATTTATCTTGTTTTTCGTTGCGGTACAAGTCCTTTAAATGGGCTCAGCTTAGAGCTATCGGAGAGGATTACTTGAATGGAATTGAGTTGGTGCAGCTTGCAGAAACCTCGGAGCACTAGAATAATGGCAGTCATCAATACGACACATATATATTCTTTCAACAAGTAAAATGCACATTTATGTTAGGAATCACTTACCTTGGTGCATGCATAAAGCAAGTCTACAATGTCAAAAAATCAATGTTGCAGGCCCCCCCAAAAGTTCCGCTCAGAAATGGTCGTGATGTTGGTATTGGGAGCCCCGTAAGAACTTTCTTTTCTCTGTCTTGGTGGTTGAAGGAGCACAGTAATAGAAAGAAGATACTTTGTAAGCGTGCCTTGTTTACCTCTACAAAAATTTAGGATGACCTTCTTAGTTTCTTTTTTTGATGTGACAGTTTTTCTTGTACGATAGTCAACCTAGCATTGTCAGTTTCTGAAAGGAAGCAATCAATACAGCAGAATGAGTCTGAACTTTTTTTACTGTACATATGGAGTGTACTTACATTTCTGAAACTTTATTGCCCATATTATTGTCTTTT-3’

NRAMP2-F 5’- GTTCTGATCGCGACAATG GC -3’

NRAMP2-R 5’- GCGGCATTATGACACAACCC -3’

11. Unigene 104920_c0

Metal transporter (NRAMP5)

5’-TGATGAGTCAAATTGTTGAAAAATAGATGAGACTGCAGCTGGAAAGCTTTCTTCAAGCAAGACAAGCGGCATGCTCCTTAATTTCCACTATAAAGAGACCACCACAACATCACTAAACAGCATGCAAGTCATGCAAAACGCAGAAATCCAGAGACATAGATAGAGAGATGGCAGGCATTCAGCAGCAGCAGCTAGTAAATGACACAGTACTGCCTGCATCACGGAATGGATCGAGCAACAGAATAGCTGCCATTAATGTGGAGGGCCATCCACAGCCTTGGGTTGATGATCTTCAGTTGGAAGACCCGGATCATCAGAAACCTGGATGGAGAAAGTTTCTATCATATGTAGGACCTGGTTTCCTTGTTTCATTGGCTTATCTAGACCCTGGAAATTTGGAAACTGATTTGCAAGCAGGAGCTAATCACAGATATGAGCTGCTATGGGTGATTCTTATCGGATTGATCTTTGCTCTCATAATCCAGTCACTTTCTGCAAACCTTGGTGTCAGCACCGGAAAGCACCTGGCAGAGCTATGCAAAGCAGAGTACCCAAAATATGTCAAGTATTGCCTGTGGTTGCTGGCAGAGATAGCCGTCATAGCTGCCGACATTCCCGAAGTGATTGGGACAGCTTTTGCGCTAAACATACTGTTTCACATCCCAGTATGGGTTGGAGTTCTTTGCACTGGTTGCAGCACCCTCCTACTCCTTGGCCTGCAGAAATATGGAGTGAGGAAGCTGGAGCTGTTAATAGCAGTGCTAGTGTTCGTTATGGCAGCATGTTTCTTTGGAGAACTGAGCTACGTAAAACCTCCTGCAACTGATGTGCTTAAGGGCATGTTTATCCCCAAGCTGTCAGGCCAGGGAGGCACCGGCGATGCCATTGCCCTACTCGGTGCCCTTATCATGCCGTAAGATCAATCCTCAAATCTGACTCAATTGCTTCGTGTTTGTAATATAAAGTCTAAGTTTTTCAATTGATGGATCTATTCTGTGCACTAAACAACTCGTTTGACTGTTTTCTCTACAGCCACAACCTCTTTCTTCACTCTGCCCTTGTTCTGTCTAGGAAAATACCAAATTCTGTGCGTGGCATCAATGTAGGCTCAAATAGAGAAACTATATATTATCTTGTAGCAGCATTTTTTTCAGGCCGGCTAACGCTTGCTCACGTGAAAATTAATAAATCCAAACATCACATTTTTTGTTTTTTGTTTTTCTACTTGCTATGCATAATTCTCCTCATGCAAATTGACCTGTATTATTTTGTTTCTTGATTTTACAGGATGCCTGTCGTTATTTTTTGATAGAGAGTGGATTCGCACTGTTGGTAGCATTTCTAATCAATCTCTCAGTCATCTCCGTATCTGGGACTGTTTGCTCAGCCCAGAATCTATCATCTGAAAATGCAGATCGGTGTGGAGATCTCACCCTTAACTCCGCCTCCTTCCTTCTTCAGAATGTGTTGGGAAAATCAAGCTCCAAGATTTATGCCATTGCTTTGTTAGCCTCAGGGCAAAGCTCCACTATTACAGGCACTTATGCAGGACAATTCATCATGCAGGTCTCTAACGAGAAGAAAAAAAAAATAGCATTTGAACTCAACTTGTCATAACTCTTAGGAGATGATAAATTACTTTCGGTTGGATGGTGTAATTTTCAGGGTTTCTTGGAACTTAAGATGAGAAAATGGATTCGGAACCTGTTGACCAGGTGCATTGCCATTGCACCTAGCCTTGTTGTCTCGATTGTTGGTGGATCATCAGGCGCAAGCCGGTTAATCATCATTGCATCGATGATTCTTTCTTTCGAATTGCCATTTGCTCTCATCCCACTTCTCAAATTCAGTAGCAGTACCACCAAGATGGGGCCACACAAGAACTCAATTTACATTATAGTAATCTCATGGATTTTGGGTCTGGGAATTATAAGCATCAATATTTATTATCTAAGCACAGGCTTTGTGGGCTGGCTACTTCACAACAATCTACCAAAAGTTGGGAACGTTTTCATCGGAATCATGGTGTTTCCTCTGATGGCAATCTATATCCTATCAGTAGCCTATTTAGCCTTCAGAAAAGACTCTGTGGAGACCTTTTTGGGGCCAAATAAGAATAACCCACATCAACAGACTAACATGGAAAAAGGACTCACCAAATCTACCGAGAACCCAGAGCTGGATCGTGTGCCATACAGAGAGGACTTGGCTGATATCCCTCTGCCAGAATAGAGGGTGTTCTCTGTCACTGGACAGGACACCACCGATCAAAGCGAGGCTAAAGCGATGTGATAATGGAGCTTTCTTGTCTGGTTGAAGTTCAAGGCATACACTAAGACTTCAGTATGCCACCTTTTGGGACTGATAACAGCAGGACTCTTCGTTTGTGAGTTCTAAGGAGTGCTCAGGTTGTAATATTGGCTTTTATTTCAGCTCCAATCGATCAGGGCCATTACTTTTGCCTTGAATTGCGAAGAAGTTTTCGTTCGTAATCAGATACTGCAGTGTAAGTTTTTGTTTTGATTTTTCTCCGCTTACAGTATTTGATTTTCATTAACTGGACCTTGGAATAAGACTACTACTAGTCTACTACTACTCTATCCGCGCTCTGCCGGTAGCCTGAGCTCGAGCGGGATCAAAAGTTATTCTACGACAACAACAAAAAAAAATATGCCTGCATTTTTCTATATGAATCGAAAAGTTGACACAATGTAGATTAAAA-3’

NRAMP5-F 5’- CTGCAAACCTTGGTGTCAGC-3’

NRAMP5-R 5’- AGCTGTCCCAATCACTTCGG-3’

12. Unigene 83604_c0

ABC transporter B family member 25 (ABCB25)

5’-CGACACAATATTTCACAACATTCATTATGGTCGTCTTTCAGCAAAGAGAGAGGAAGTCTATGATGCTGCCCAACATGCTGCAATCCATGACACTGTCATGAACTTCCCCAATAAATATTTTACAATAGTAGGAGAACGAGGGCTCAAGTTAAGTGGTGGTGAGAAGCAGCGTGTGGCTTTGGCCCGTGCATTTTTGAAAGCCGCTCCTATCATGCTATGTGATGAAGCTACAAGTG-3’

ABCB25-F 5’- CTGCCCAACATGCTGCAATC-3’

ABCB25-R 5’- AGCATGATAGGAGCGGCTTT-3’

13. Unigene 103730_c0

ABC transporter G family member 36 (ABCG36)

5’-TTTTTTAAAATGAGAATTGTTAATAAGATTTAATAACCTTTACATTTATATATATGAAACGAAAAATGCTACATTGTCTTCATTGTTGATCCCAGCAATAATTTTTCCCAAGTCGTCTATAAGATTTACTAATCCCTTACATACTTGTTTGCAAAACAAATGTGCAACATTCTATGAAGAACGACAATCCGTATACAAAGATTACCTTATACTAAACTTTAATGCGTATGGTCTCTACTACATATATATATATCTACAAGCTCAACTCTTACAGATCTTGCTTCAAGAAATTAAAGTACGAGTTTGATAAATCTCTCCTATCTTGTCTGGAAGTTCAGTGTCCTTATGCAGAAGGCAAACAAGAAAGCAAAGAAGACGGTGAAGCTAATCAAGACTGCGGCAACCTGCCCCATGAAATCTGGGTCATATCCATAATATTCTTGTATATAATTCTTGATTATTACAGGATTTGGGCGACCAGGAACTTCAATGGTGTCCATGACATCACCATACTGGGACACGATTAGTCCGTACACTGTCCACGCCACAGGGCAAATCCAATAGTACCAGACCCACCACTTGGGAATTCTCGGTCTTGGTATAAAGAAGCCAGAGAAAAGATTGAAGAGGGAATAGAACATTGCTGCAAAAATGGCTGCTACTTGGTGGTTTGGGGTGACGGAAACAGTCATCATTCCATAATATGTGAAGTAAAGGAAGGAGAAGAAGTTGACAAAGAAGAACCACAAGAATTTGACCGCAGTCCACTCAAATCCCACCATCGCATACACGATAAGCGTGTAGTATGTAGTTTGAACAAATACGTACGGTATTTCACAAACAACCTGTGCGATGGCATAAGGTAATGCAGAATACATTCCGGCTGCTTTTTCTCGATAAAACACCGTTCTTTCGACCGCAACTACTGGCTGTACAGTTGAGCAGTTGTTAATACCGATGAACAAGACTGAAGCATACATGGCACCGATGATCATATTCAGATCACTCGAGCTATCCCTTTTTGTTCCAATCTTCCAGAATATGCTACCGATCATGAGAGCACAAACCAAGGTGAAGAAGTATCTAACAAGATTGTAATCGGGACTTCTCCAGTAGGTCCACCATTGCTTCCACAGGCAAGATTTGAACTGCCCCCATACAGATTCTGAATACTGAGTGGCGAAATAAAGGTCTTTTGCTCCTGCTGGCGGCGTGCTCAACTCCTTCACCAAAGCCTTGTTTCTCTGGTGCAGGGATGAAGCTTTGTGGTGTTCCGCAAAGTCCATTCCAAGCCTGACTTCGGCGGCAACCGAACTCACTTCTAGCATCCATGTCGCTGGATTGTACTTTTCTTTGATTTTAGGGACTCCAGGAATGGCCTCGAAGTATTCAACGATCTTGTGAGAATTTCGACCCAATGGTCCTGAGTAGATTACTTGTCCTCCTCTCTTCATTAACAGCAATTCATCAAAGGCTTCGAAGATGTCAATGCTAGGTTGATGGATCGTACAGACAACTGTTCTCCCAGTATCCACAGTGTTTCTCACAGTCCTCATAACAATGGCAGCTGCCCTTGCATCAAGACCAGATGTTGGTTCGTCCATGAAAATGATGGAGGGATTAGCAACCAGCTCCACTGCTATTGTTAACCTCTTTCTCTGTTCCGTCGACAACCCGGTGATTCCTGGAAGCCCAACTACAGAATCCTTGAGATTGTTTAGCTCGACCAACTCCATCACTTCATCCACAAAAATCATCTTTTCTTGTTTGCTGACTTCTTTAGGGAGACGAAGGAATGCTGAATAAATCAATGATTCTTTAACAGTGACTTGAGGAGAGTGGATATCATTCTGTTCGCAATATCCAGAAACTCTTGCAAACGTTTCCTGTTTCTTGGAGAACCCGGAAATTCTAATGTCACCTTCAACGTATCCACCAGTCTTTCTTCCTGCCAAAACATCCATCAGTGTGGTCTTTCCTGCTCCACTGACTCCCATTAATGCTGTGAGCACTCCAGGCCTAAATGCTCCTGTTACTTCTCGAAGCAGTTGCAGCCTATCCTCTGCAACTCCTTGCTCCTTCATTTCCGCGGGCATGTCCACAAAATAATTCACACTGTCAAAGGACATGGCTAGAGGAGTGAAAGGAAGAACCATTCCTCTCTTGTGAGCAACTCCATTCGCTGCCTCAAACGAATCGGCATTTCCACCTAGTGCATCGGGATTGGATCGGCTGTTCATTCTCAAGATTGCCATTTCACCTGTATTGTTTCCGTCAGAATGGGATAATGACTGTGTTGACCTGGTTTCTTCTTTTGTTGTTTCCTCAGTGATCATGGCCTGTGACTTTCCAGCAGGACTAAGGTACGCGAGGGCAAAGGTGAAGAGAATATTGAATAGAACAGCAAAGCCCAGAATAGCAGCTGTACCAATCCAATACCAGTTCTTATCCGTGTAAACATCGAAGTTCTTGAGCACTGCTGTGCCTAAACTGATAGTGGTGTTTGTACCCGGTTTGTTCATCCACCTTGGCGCAGACATTTCGTTCCCAGCTATGGCATTGAAGCCATAAGACAGTGGTGAAACCCAGTAACCCCATCCCCACCAATCTGGAATGGCACCTTTAGGTACGATGAAACCTCCGAGCAAGAAAATAAGCAGTAGAGTGAGAGCCCCGCCGGTGTTGGCAATGATCATGGTTCTGCAGACCCCAGCAATGAGCCTAAAGAGCCCAGAAGCCATCTGCTGGATGAAAAATACCAGTAGCAGCTGCTTGAAAAACCTGCTAGCTTCGGGTGCAAAACCAACGGAGTAATAGGTAATCGACACCCAAACAACAGATTCAATTATAGACATTGGCAGCTGTAGCAGGAAAGTTGGCAGAGTGAAAGTCCAGGCAGGATGGAATTGGAGATCTCTTTGCTTGTAGAATACAGGAAGCCTCTTGATTATAAGCGAGAGCTCAGCAAAACCATTGAACATGTTAATGATCATTGTAAACAGAAGGGCACCGACATATACGGCTCCATCTTCTTCATTCCTCGTGTGCATTTTAGACTTGATGAACACCGTGGACATTATAATTGCCATAATAATAAGCTGGACCGTCTTGGCCACGTAAACATACGAGTTTCTCTGAACCAATATCCATTCTCTGTCCCAACATGCCCTGAGCAGCTCCATTTTAGGAACAGAATACTTCGAGAATGATAGAGCTGCTTTGTGGCCTTGCGTCTTGTCAAATGGAACAGAGAGTTCGTTCTCCAGCCTCATCCCCACATGAAACCTCTTGAATCTTTCGACAAATTCTGGGACTGACACGTATCTATAGGGCCTGTTTCTATCATCCCAGTACTGTTCTTGGTCTTTCTTCGACGTAACCTCTTGTAAGAAATCAGCTGTGCCCTTTCTCTCAGGACAGCGGAACCCACAGCTCTCGAAGAAAGCAAGAATGTGCTCTCGTGGGCCCTGGTATACAATCTGGCCTTCTGATAAAAGGATGATATCATCAAAGAGATCGAACGTCTCAGGAGCAGGCTGGAGCAGGGACACCAAGATTGTAGCCTCAGTGTAGTGTACAATATGCTGCAAGCACTTCACTATTTGATACGTCGTGGAGCTATCTAGACCCGTTGATATCTCATCCATGAATAGAGTTTTTGTGGGCCCGACAATCATCTCTCCTGTAGTCACTCGTTTTTTCTGTCCGCCCGATATCCCTCGTATCATTTCGTCTCCAACGATGGTATCCTTGCATATATCAAGTCCTAATATTTTGAGTGTGTAGTCAGTAATCAGACTGCTTTCAACTCCTTCCATTGCAGTTGCCTTCATGAAAAGATCCACTTCTGCCTCTGGAAATATTCCAGCATCCTTCTCTCTTCTTGCAAGCTCGCTTAGAAGATCATATCGTGTCCCGACCCCCTGACACCTTGCTGAGAAATCTAGGGTTTCTTTCACAGTCATTTCTCCTATGTGAACATCATTTTGGCTGATATATGCTGATGATTTCCGTGGCATAAACTCCTTGAGCTCATATCCATTATATGTCAGGTCCCCTGTAACCTTAAGACTCGGGTCCAACTTTCCCGCAAGAGCCAGCAAAAGAGTAGTTTTTCCAGAGGATGGTGGACCTAGCAACAGTGCCATCCGTGATGGTTTTATAATCCCAGATGCATCGTTGAGGATTCTGAGCTTCGTTCTGGCAGCCAAATTTATCCCAATCACACCAAGAGCTGATTCAACCATGTTTCTTGCAGCGTTTGGGAGGGTCGGGAGAGCTCTGGAGCCAATGTGGCAGTCAGCTTCGATTGTTAGATGATCGAACCTAACCTCGATGGTCGGGAGCCGGATACCAACCTTCTCAACTCTTTGTCTGAATTTTTTCAAGAATTTTTCGTTATCTTCCTCTGCAACCTTGAAGAGCTTGTCGATGAAATTCTGTCTGTCATTTACGTCAAGCTTTCTCACATCAACTTCTTTATGCAGCAGCGTCTTATTACCCTGGACCTCAGTCTCCGCAAAAGATTTGATGATACTTGTTCTTAACCTATTGTATGTCGGCAACTTCTCTATGGCAGCCCATTTGAGAGCTTCTTCATCTTCATCGACCAGGCTGCTTCTCCTGGATTGCCTGCCAACTGAAAACATGTCTTCCATGTTCCAACTAGCCCTGCTCAAGCTCCTGCTTATGCTCCTGCTCAAGTTGCTGTGACTCGGTCGTCTACCAGAAGCTCGAGCTCTCTCTACACCATCCATCTTTCTCTTATTCCTCTGCTGTTCCTTGAAAAAAAAAAAAAAGAGTTGTGGAACGAGAGACGGGGATGAGGTTGGTGGAGTTTTGAAAGTTCCCTTCTCACTTGGTCACCAAGAAAATCTTGAGAAGTTCTCCCCCCCTCTCTTCCCTTTTAAACGAGAATGGTG-3’

ABCG36-F 5’- CTTGCTCCTTCATTTCCGCG-3’

ABCG36-R 5’- TTGAGGCAGCGAATGGAG TT-3’

14. Unigene 732621_c0

ABC transporter G family member 35 (ABCG35)

5’-ATATGCTGCAAGCACTTCACTATCTGGTATGTTGTGGAGCTATCCAGACCGGTCGATATCTCATCCATGAATAGTGTTTTTGTGGGCCCAACGATCATCTCTCCTGTTGTCACTCGCTTTTTCTGTCCTCCTGATATTCCTCGTATCATTTCATCTCCAACAACGGTGTCTTTGCATATATCTAGTCCTAGTATTTTGAGTGTGTAGTCAGTGATAAGACTGCTCTCAACTCCTTCCATTGCTGTTGCCTTCATGAAAAGATCCACCTCTGCCTCTGGAAATATTCCAGCGTCCTTTTCTCTTCTTGCAAGTTCACTTAGGAGATCATATCGGGTCCCG-3’

ABCG35-F 5’- TTTGTGGGCCCAACGATCAT -3’

ABCG35-R 5’- GGCAACAGCAATGGAAGGAG -3’

15. Unigene 95863_c0

Glutathione gamma-glutamylcysteinyl transferase 1 (PCS1)

5’-TTTTACAATTAACTTTTAATTAGTTCATTTACTGAAAAAAGCTAATAAAATATTTCATGAATACTAATCTACTTCAACATTATCTAACTATTTCAACAACTTTCATCTCTCACCACATCCATCACTATCACCGTCGTCGTCATCTCATCTCCAACCCCCCTCTATCATCACTATTGAATAATATTTTACATGTAAAATATGAGCATTTTTCAAGCTAAACAATGAAAAATATTTTATGAAAAACAAAGGAAACACTTAGGAGAAGAAAATATGATTTTTGAAAGACTATCTTGAGCTCGCCCGGGGCAAACAACAAATGATCAAGACGCTCGAGCTCATTGTGATATTCTTTCATACCACTTTCAAAACATACATGACCAGCTGCTGTAAGGTACAAGCAAGACTTTTGAATGATTAGTGACACCGCATAATAAATCAGAAAACTCAGTAAATTGGTGAGAAATATACTCCACGATAATAGACAGTGTGCCCTGCTTACTCAGTCAGCATGTTAATTGATTCAAGTTTTGAACAGATTCAGTCAACGAACTTGCCTCTTACCTCTAGATCAACCAGGAGCACTGATAAGCCTCGCCTACCATGAGTTTTGGTGTTTGGATGACAAATGCAGCTCCTCTACGAGGTCATTTCACTCCTAGGGCTTTGGTGGGTTATAGATCTTCCATGGGTAGGTACACTTGGCGTTAAGACAATGAAGTTACAGAGAACAGGTACAAGAAAATGCTACGAAAGAGGTGCGTCAAGATCCTCATCTACCCTATTCTCTTGGCATCTTTTGAGGAGGTAGAGCTGACGTCGTAGATGCAGAACCTCTTCTTGAAGCATAACTGGGAGATATTCAGTTGAAACAAGAGTAGATATTTCTTGCAAAAGTTTCTCATCTTTGATACCAGACCATGTTTCTGGAGGCAAAGCCAATAAAAGCGCTGTTAAAACATCATTGCCAGCTGGGTACATCCCAATGTGACTGCTTGGACCACAGCCACAGCAATTCATTTTTGTTTGAGATGAAGGAACCAGCACATCAAGCCCCTGCTCACTGATACCATCTACCACTGTCCCAGAAACCAAAGTAATAGGCTTTTCACCATTAGTTTTCAAGCATTTCACACATTTTTCCTTACAGCAATACCCTTCCACAGAAGAGGACTTCCCAGCCAAAATCTGTGCCCCCTGGCAACAGACACTTGCAGCAATATCAGGCAGATCATCTTCTTGACCCAGAATTGGTGCATTTCTGCAACATGAATTTGATGAAGACAAAAATGCCACAACACGTTTAAAAAGGCCGGTCTCCTGCACCTGTTTCAACACCTCTTCCTTGACAGAAAGCCTTCCTTTCTCCTCTTGGCTTAGACTTTGACCACCATCCTCTTGTCTCCGAACTTCTGCGACCCACTTGATGAAATCCCCAAAATTTGATGGGAGTGATAGGAAAACAACACGGAGAACTTTGTTGATATCTTTTACATCCTCTGACTTTAAGAGAAGAGGAACATCATCCATTAAATACTTCGCAACACCAACCCAACTCTCATGCTTGCAGCTCAGGGTATAAAGCAGTCCTGGCTCTGTGTGAGGCCTAGATATTAGCATGAACCCTCTTCGTTGAGAAGTTGTCTCGTCAGTCCTGTCCATTGCTTCCCAAAGAAGTGTTAGTGGAACCCAATGAGGAGGATACTTGAACCGCGCAACATCTAAAATCAGTGCCATGTCCTTTCCAGCATGATAACCACCAATAGGAGAAAAATGACCAGTTCCTGTCTGGAAAAAAAAAAGTTCCCGAAAACTTCCTGTGCTAAATTAAGCAGGGAGTACTAAATTTATGGTGTGAAGCATTTAAGCAACAAATTAATGAAGTAAAATATCGATGTTAGTGTTATTTTCCTATTACACACCAAAATAACAAAAACATCAGAGGCAATGTAGATCCACCATGTTCTACTATTAAATCTTTAACGAATGAAGCAAAAAGTAACATAGCAAGATTTTTTTTGGATTCATTAGAAAGTAGACAATTATGAAAAAGATTCAATCAAGTTAAAGATGAATACCTGTTTAAAAGTTCCTCTATGATAAGACGAGATCAAATGGCAATCATCAGAAGTTGAACATCTCATAATGTGTACACGGAAGTCATCTAAAGTGCTTTGATTTGTTCGGTATGCTTGAACTTTAACCCCGGCACAATGAGCCAGACACACTAGCTTCCCAAATGAAATACCCTTAGCTTTAACCTGTTCTAAGGGCTCACAGCAGTCTAGCATAGATTCATCAAACCACCTCCAAGATCCTTTCCATTTCCTCCCAGGGTCAATAGCTAGGGCATTCAGGACCATAGAAAGACTTGCCAAACCACAATACGCAGGCTCCGACTGTGTCTGGAAGTAAGAAATCAACCTATAAAAACCTTCCATCGTTCCATTTTGAATTGCTTCCATAAAAAGTTGCTTTCCTTCGGCGGAAGCGAAATCGATGGCAGGTGGAGAAGGGAGGATTCGACGGTAGAATCCAGCCATGGCCATTTTTTCACTTTTCTAAAAACTAGTCGATATCAGTAATAAATTGTGTGTGTGTTTTGGGAAATCGATGGCTATAAAAACAGAAAAAAA-3’

PCS1-F 5’- CGTCAGTCCTGTCCATTG CT -3’

PCS1-R 5’- ATG CTG GAAAGG ACATGGCA -3’
