# Supplementary material for: Transcriptome Sequencing and Expression Analysis of Cadmium (Cd) Transport and Detoxification Related Genes in Cd-Accumulating Salix integra
Source: Front Plant Sci. 2016 Oct 28;7:1577. doi: 10.3389/fpls.2016.01577 (PMC5083712; doi:10.3389/fpls.2016.01577)
Supplement: File S2 — Pathway assignment based on KEGG. [file DataSheet2.DOC]

File S2 Pathway assignment based on KEGG

|  | Pathway | Pathway Id | Gene_number  (11,680) | Percentage |
| --- | --- | --- | --- | --- |
| 1 | Ribosome | ko03010 | 587 | 5.03% |
| 2 | Protein processing in endoplasmic reticulum | ko04141 | 382 | 3.27% |
| 3 | Oxidative phosphorylation | ko00190 | 350 | 3.00% |
| 4 | RNA transport | ko03013 | 300 | 2.57% |
| 5 | Purine metabolism | ko00230 | 269 | 2.30% |
| 6 | Spliceosome | ko03040 | 266 | 2.28% |
| 7 | Glycolysis / Gluconeogenesis | ko00010 | 254 | 2.17% |
| 8 | Plant hormone signal transduction | ko04075 | 216 | 1.85% |
| 9 | Ubiquitin mediated proteolysis | ko04120 | 215 | 1.84% |
| 10 | Pyrimidine metabolism | ko00240 | 206 | 1.76% |
| 11 | Phagosome | ko04145 | 203 | 1.74% |
| 12 | Endocytosis | ko04144 | 197 | 1.69% |
| 13 | Starch and sucrose metabolism | ko00500 | 186 | 1.59% |
| 14 | Pyruvate metabolism | ko00620 | 184 | 1.58% |
| 15 | Ribosome biogenesis in eukaryotes | ko03008 | 177 | 1.52% |
| 16 | RNA degradation | ko03018 | 174 | 1.49% |
| 17 | mRNA surveillance pathway | ko03015 | 170 | 1.46% |
| 18 | Lysosome | ko04142 | 161 | 1.38% |
| 19 | Peroxisome | ko04146 | 157 | 1.34% |
| 20 | Amino sugar and nucleotide sugar metabolism | ko00520 | 152 | 1.30% |
| 21 | Arginine and proline metabolism | ko00330 | 150 | 1.28% |
| 22 | Cysteine and methionine metabolism | ko00270 | 145 | 1.24% |
| 23 | Fatty acid metabolism | ko00071 | 141 | 1.21% |
| 24 | Citrate cycle (TCA cycle) | ko00020 | 138 | 1.18% |
| 25 | Glycerophospholipid metabolism | ko00564 | 134 | 1.15% |
| 26 | Carbon fixation in photosynthetic organisms | ko00710 | 131 | 1.12% |
| 27 | Glutathione metabolism | ko00480 | 128 | 1.10% |
| 28 | Valine, leucine and isoleucine degradation | ko00280 | 122 | 1.04% |
| 29 | Alanine, aspartate and glutamate metabolism | ko00250 | 120 | 1.03% |
| 30 | Aminoacyl-tRNA biosynthesis | ko00970 | 117 | 1.00% |
| 31 | Propanoate metabolism | ko00640 | 112 | 0.96% |
| 32 | Proteasome | ko03050 | 110 | 0.94% |
| 33 | Plant-pathogen interaction | ko04626 | 109 | 0.93% |
| 34 | Inositol phosphate metabolism | ko00562 | 108 | 0.92% |
| 35 | DNA replication | ko03030 | 107 | 0.92% |
| 36 | Nucleotide excision repair | ko03420 | 107 | 0.92% |
| 37 | Pentose phosphate pathway | ko00030 | 106 | 0.91% |
| 38 | Glycerolipid metabolism | ko00561 | 106 | 0.91% |
| 39 | Fructose and mannose metabolism | ko00051 | 104 | 0.89% |
| 40 | Protein export | ko03060 | 100 | 0.86% |
| 41 | Glycine, serine and threonine metabolism | ko00260 | 99 | 0.85% |
| 42 | Nitrogen metabolism | ko00910 | 98 | 0.84% |
| 43 | Phosphatidylinositol signaling system | ko04070 | 98 | 0.84% |
| 44 | Photosynthesis | ko00195 | 94 | 0.80% |
| 45 | Phenylalanine metabolism | ko00360 | 94 | 0.80% |
| 46 | N-Glycan biosynthesis | ko00510 | 93 | 0.80% |
| 47 | Wnt signaling pathway | ko04310 | 92 | 0.79% |
| 48 | Tryptophan metabolism | ko00380 | 89 | 0.76% |
| 49 | beta-Alanine metabolism | ko00410 | 89 | 0.76% |
| 50 | Pentose and glucuronate interconversions | ko00040 | 85 | 0.73% |
| 51 | Porphyrin and chlorophyll metabolism | ko00860 | 85 | 0.73% |
| 52 | Terpenoid backbone biosynthesis | ko00900 | 82 | 0.70% |
| 53 | Phenylpropanoid biosynthesis | ko00940 | 74 | 0.63% |
| 54 | Basal transcription factors | ko03022 | 73 | 0.63% |
| 55 | Glyoxylate and dicarboxylate metabolism | ko00630 | 72 | 0.62% |
| 56 | ABC transporters | ko02010 | 72 | 0.62% |
| 57 | Tyrosine metabolism | ko00350 | 71 | 0.61% |
| 58 | Homologous recombination | ko03440 | 71 | 0.61% |
| 59 | Mismatch repair | ko03430 | 70 | 0.60% |
| 60 | Lysine degradation | ko00310 | 69 | 0.59% |
| 61 | RNA polymerase | ko03020 | 69 | 0.59% |
| 62 | Galactose metabolism | ko00052 | 68 | 0.58% |
| 63 | Butanoate metabolism | ko00650 | 68 | 0.58% |
| 64 | Fatty acid biosynthesis | ko00061 | 65 | 0.56% |
| 65 | Phenylalanine, tyrosine and tryptophan biosynthesis | ko00400 | 64 | 0.55% |
| 66 | Biosynthesis of unsaturated fatty acids | ko01040 | 61 | 0.52% |
| 67 | Sphingolipid metabolism | ko00600 | 59 | 0.51% |
| 68 | Base excision repair | ko03410 | 59 | 0.51% |
| 69 | Ascorbate and aldarate metabolism | ko00053 | 58 | 0.50% |
| 70 | Valine, leucine and isoleucine biosynthesis | ko00290 | 57 | 0.49% |
| 71 | Histidine metabolism | ko00340 | 51 | 0.44% |
| 72 | Ubiquinone and other terpenoid-quinone biosynthesis | ko00130 | 49 | 0.42% |
| 73 | Cyanoamino acid metabolism | ko00460 | 49 | 0.42% |
| 74 | Pantothenate and CoA biosynthesis | ko00770 | 49 | 0.42% |
| 75 | Carotenoid biosynthesis | ko00906 | 48 | 0.41% |
| 76 | Other glycan degradation | ko00511 | 46 | 0.39% |
| 77 | Sulfur metabolism | ko00920 | 45 | 0.39% |
| 78 | TGF-beta signaling pathway | ko04350 | 45 | 0.39% |
| 79 | Progesterone-mediated oocyte maturation | ko04914 | 44 | 0.38% |
| 80 | mTOR signaling pathway | ko04150 | 43 | 0.37% |
| 81 | One carbon pool by folate | ko00670 | 42 | 0.36% |
| 82 | Steroid biosynthesis | ko00100 | 40 | 0.34% |
| 83 | Notch signaling pathway | ko04330 | 40 | 0.34% |
| 84 | Regulation of autophagy | ko04140 | 38 | 0.33% |
| 85 | Photosynthesis - antenna proteins | ko00196 | 37 | 0.32% |
| 86 | Lysine biosynthesis | ko00300 | 37 | 0.32% |
| 87 | Selenocompound metabolism | ko00450 | 37 | 0.32% |
| 88 | SNARE interactions in vesicular transport | ko04130 | 36 | 0.31% |
| 89 | Dorso-ventral axis formation | ko04320 | 36 | 0.31% |
| 90 | Natural killer cell mediated cytotoxicity | ko04650 | 36 | 0.31% |
| 91 | Ether lipid metabolism | ko00565 | 33 | 0.28% |
| 92 | Taurine and hypotaurine metabolism | ko00430 | 31 | 0.27% |
| 93 | Hedgehog signaling pathway | ko04340 | 31 | 0.27% |
| 94 | Arachidonic acid metabolism | ko00590 | 30 | 0.26% |
| 95 | Circadian rhythm - plant | ko04712 | 28 | 0.24% |
| 96 | alpha-Linolenic acid metabolism | ko00592 | 26 | 0.22% |
| 97 | Limonene and pinene degradation | ko00903 | 26 | 0.22% |
| 98 | Insulin signaling pathway | ko04910 | 26 | 0.22% |
| 99 | Flavonoid biosynthesis | ko00941 | 25 | 0.21% |
| 100 | Glycosylphosphatidylinositol(GPI)-anchor biosynthesis | ko00563 | 24 | 0.21% |
| 101 | Folate biosynthesis | ko00790 | 24 | 0.21% |
| 102 | Tropane, piperidine and pyridine alkaloid biosynthesis | ko00960 | 23 | 0.20% |
| 103 | Drug metabolism - other enzymes | ko00983 | 23 | 0.20% |
| 104 | Cell cycle | ko04110 | 22 | 0.19% |
| 105 | Drug metabolism - cytochrome P450 | ko00982 | 21 | 0.18% |
| 106 | Jak-STAT signaling pathway | ko04630 | 21 | 0.18% |
| 107 | Glycosphingolipid biosynthesis - globo series | ko00603 | 20 | 0.17% |
| 108 | Thiamine metabolism | ko00730 | 19 | 0.16% |
| 109 | Nicotinate and nicotinamide metabolism | ko00760 | 19 | 0.16% |
| 110 | Zeatin biosynthesis | ko00908 | 19 | 0.16% |
| 111 | Synthesis and degradation of ketone bodies | ko00072 | 18 | 0.15% |
| 112 | Riboflavin metabolism | ko00740 | 18 | 0.15% |
| 113 | Oocyte meiosis | ko04114 | 18 | 0.15% |
| 114 | Sulfur relay system | ko04122 | 18 | 0.15% |
| 115 | ECM-receptor interaction | ko04512 | 18 | 0.15% |
| 116 | Fatty acid elongation in mitochondria | ko00062 | 17 | 0.15% |
| 117 | Glycosaminoglycan degradation | ko00531 | 17 | 0.15% |
| 118 | Isoquinoline alkaloid biosynthesis | ko00950 | 17 | 0.15% |
| 119 | Circadian rhythm - mammal | ko04710 | 17 | 0.15% |
| 120 | Diterpenoid biosynthesis | ko00904 | 16 | 0.14% |
| 121 | Tight junction | ko04530 | 16 | 0.14% |
| 122 | Antigen processing and presentation | ko04612 | 16 | 0.14% |
| 123 | MAPK signaling pathway | ko04010 | 15 | 0.13% |
| 124 | Chloroalkane and chloroalkene degradation | ko00625 | 14 | 0.12% |
| 125 | Metabolism of xenobiotics by cytochrome P450 | ko00980 | 14 | 0.12% |
| 126 | Focal adhesion | ko04510 | 14 | 0.12% |
| 127 | Benzoate degradation | ko00362 | 13 | 0.11% |
| 128 | Vitamin B6 metabolism | ko00750 | 13 | 0.11% |
| 129 | Non-homologous end-joining | ko03450 | 13 | 0.11% |
| 130 | Pathways in cancer | ko05200 | 13 | 0.11% |
| 131 | Glycosphingolipid biosynthesis - ganglio series | ko00604 | 12 | 0.10% |
| 132 | Methane metabolism | ko00680 | 12 | 0.10% |
| 133 | Neuroactive ligand-receptor interaction | ko04080 | 12 | 0.10% |
| 134 | Mucin type O-Glycan biosynthesis | ko00512 | 11 | 0.09% |
| 135 | PPAR signaling pathway | ko03320 | 10 | 0.09% |
| 136 | Regulation of actin cytoskeleton | ko04810 | 10 | 0.09% |
| 137 | Melanogenesis | ko04916 | 10 | 0.09% |
| 138 | Bacterial secretion system | ko03070 | 9 | 0.08% |
| 139 | MAPK signaling pathway - fly | ko04013 | 9 | 0.08% |
| 140 | Phototransduction - fly | ko04745 | 9 | 0.08% |
| 141 | Adipocytokine signaling pathway | ko04920 | 9 | 0.08% |
| 142 | Alzheimer's disease | ko05010 | 9 | 0.08% |
| 143 | Chagas disease (American trypanosomiasis) | ko05142 | 9 | 0.08% |
| 144 | Hepatitis C | ko05160 | 9 | 0.08% |
| 145 | Other types of O-glycan biosynthesis | ko00514 | 8 | 0.07% |
| 146 | Aminobenzoate degradation | ko00627 | 8 | 0.07% |
| 147 | Biotin metabolism | ko00780 | 8 | 0.07% |
| 148 | Retinol metabolism | ko00830 | 8 | 0.07% |
| 149 | Two-component system | ko02020 | 8 | 0.07% |
| 150 | ErbB signaling pathway | ko04012 | 8 | 0.07% |
| 151 | Calcium signaling pathway | ko04020 | 8 | 0.07% |
| 152 | Vascular smooth muscle contraction | ko04270 | 8 | 0.07% |
| 153 | Adherens junction | ko04520 | 8 | 0.07% |
| 154 | NOD-like receptor signaling pathway | ko04621 | 8 | 0.07% |
| 155 | Neurotrophin signaling pathway | ko04722 | 8 | 0.07% |
| 156 | Prostate cancer | ko05215 | 8 | 0.07% |
| 157 | Naphthalene degradation | ko00626 | 7 | 0.06% |
| 158 | Stilbenoid, diarylheptanoid and gingerol biosynthesis | ko00945 | 7 | 0.06% |
| 159 | Chemokine signaling pathway | ko04062 | 7 | 0.06% |
| 160 | Cardiac muscle contraction | ko04260 | 7 | 0.06% |
| 161 | Geraniol degradation | ko00281 | 6 | 0.05% |
| 162 | C5-Branched dibasic acid metabolism | ko00660 | 6 | 0.05% |
| 163 | Lipoic acid metabolism | ko00785 | 6 | 0.05% |
| 164 | Apoptosis | ko04210 | 6 | 0.05% |
| 165 | VEGF signaling pathway | ko04370 | 6 | 0.05% |
| 166 | Toll-like receptor signaling pathway | ko04620 | 6 | 0.05% |
| 167 | Leukocyte transendothelial migration | ko04670 | 6 | 0.05% |
| 168 | GnRH signaling pathway | ko04912 | 6 | 0.05% |
| 169 | Salivary secretion | ko04970 | 6 | 0.05% |
| 170 | Gastric acid secretion | ko04971 | 6 | 0.05% |
| 171 | Huntington's disease | ko05016 | 6 | 0.05% |
| 172 | Amoebiasis | ko05146 | 6 | 0.05% |
| 173 | D-Glutamine and D-glutamate metabolism | ko00471 | 5 | 0.04% |
| 174 | Caprolactam degradation | ko00930 | 5 | 0.04% |
| 175 | Gap junction | ko04540 | 5 | 0.04% |
| 176 | Long-term potentiation | ko04720 | 5 | 0.04% |
| 177 | Long-term depression | ko04730 | 5 | 0.04% |
| 178 | Bile secretion | ko04976 | 5 | 0.04% |
| 179 | Parkinson's disease | ko05012 | 5 | 0.04% |
| 180 | Toxoplasmosis | ko05145 | 5 | 0.04% |
| 181 | Steroid hormone biosynthesis | ko00140 | 4 | 0.03% |
| 182 | Glycosaminoglycan biosynthesis - heparan sulfate | ko00534 | 4 | 0.03% |
| 183 | Linoleic acid metabolism | ko00591 | 4 | 0.03% |
| 184 | Brassinosteroid biosynthesis | ko00905 | 4 | 0.03% |
| 185 | Flavone and flavonol biosynthesis | ko00944 | 4 | 0.03% |
| 186 | Renin-angiotensin system | ko04614 | 4 | 0.03% |
| 187 | Olfactory transduction | ko04740 | 4 | 0.03% |
| 188 | Glioma | ko05214 | 4 | 0.03% |
| 189 | Chlorocyclohexane and chlorobenzene degradation | ko00361 | 3 | 0.03% |
| 190 | Styrene degradation | ko00643 | 3 | 0.03% |
| 191 | Bacterial chemotaxis | ko02030 | 3 | 0.03% |
| 192 | MAPK signaling pathway - yeast | ko04011 | 3 | 0.03% |
| 193 | Axon guidance | ko04360 | 3 | 0.03% |
| 194 | Osteoclast differentiation | ko04380 | 3 | 0.03% |
| 195 | T cell receptor signaling pathway | ko04660 | 3 | 0.03% |
| 196 | Fc gamma R-mediated phagocytosis | ko04666 | 3 | 0.03% |
| 197 | Proximal tubule bicarbonate reclamation | ko04964 | 3 | 0.03% |
| 198 | Pancreatic secretion | ko04972 | 3 | 0.03% |
| 199 | Protein digestion and absorption | ko04974 | 3 | 0.03% |
| 200 | Fat digestion and absorption | ko04975 | 3 | 0.03% |
| 201 | Rheumatoid arthritis | ko05323 | 3 | 0.03% |
| 202 | Arrhythmogenic right ventricular cardiomyopathy (ARVC) | ko05412 | 3 | 0.03% |
| 203 | Dilated cardiomyopathy | ko05414 | 3 | 0.03% |
| 204 | Primary bile acid biosynthesis | ko00120 | 2 | 0.02% |
| 205 | Caffeine metabolism | ko00232 | 2 | 0.02% |
| 206 | Bisphenol degradation | ko00363 | 2 | 0.02% |
| 207 | Novobiocin biosynthesis | ko00401 | 2 | 0.02% |
| 208 | Peptidoglycan biosynthesis | ko00550 | 2 | 0.02% |
| 209 | Flagellar assembly | ko02040 | 2 | 0.02% |
| 210 | Cytokine-cytokine receptor interaction | ko04060 | 2 | 0.02% |
| 211 | p53 signaling pathway | ko04115 | 2 | 0.02% |
| 212 | RIG-I-like receptor signaling pathway | ko04622 | 2 | 0.02% |
| 213 | Hematopoietic cell lineage | ko04640 | 2 | 0.02% |
| 214 | B cell receptor signaling pathway | ko04662 | 2 | 0.02% |
| 215 | Phototransduction | ko04744 | 2 | 0.02% |
| 216 | Aldosterone-regulated sodium reabsorption | ko04960 | 2 | 0.02% |
| 217 | Vasopressin-regulated water reabsorption | ko04962 | 2 | 0.02% |
| 218 | Collecting duct acid secretion | ko04966 | 2 | 0.02% |
| 219 | Amyotrophic lateral sclerosis (ALS) | ko05014 | 2 | 0.02% |
| 220 | Bacterial invasion of epithelial cells | ko05100 | 2 | 0.02% |
| 221 | Vibrio cholerae infection | ko05110 | 2 | 0.02% |
| 222 | Leishmaniasis | ko05140 | 2 | 0.02% |
| 223 | Basal cell carcinoma | ko05217 | 2 | 0.02% |
| 224 | Hypertrophic cardiomyopathy (HCM) | ko05410 | 2 | 0.02% |
| 225 | Penicillin and cephalosporin biosynthesis | ko00311 | 1 | 0.01% |
| 226 | Fluorobenzoate degradation | ko00364 | 1 | 0.01% |
| 227 | Glycosaminoglycan biosynthesis - chondroitin sulfate | ko00532 | 1 | 0.01% |
| 228 | Xylene degradation | ko00622 | 1 | 0.01% |
| 229 | Toluene degradation | ko00623 | 1 | 0.01% |
| 230 | Polycyclic aromatic hydrocarbon degradation | ko00624 | 1 | 0.01% |
| 231 | Nitrotoluene degradation | ko00633 | 1 | 0.01% |
| 232 | Ethylbenzene degradation | ko00642 | 1 | 0.01% |
| 233 | Phosphotransferase system (PTS) | ko02060 | 1 | 0.01% |
| 234 | Cell cycle - yeast | ko04111 | 1 | 0.01% |
| 235 | Cell adhesion molecules (CAMs) | ko04514 | 1 | 0.01% |
| 236 | Complement and coagulation cascades | ko04610 | 1 | 0.01% |
| 237 | Cytosolic DNA-sensing pathway | ko04623 | 1 | 0.01% |
| 238 | Fc epsilon RI signaling pathway | ko04664 | 1 | 0.01% |
| 239 | Circadian rhythm - fly | ko04711 | 1 | 0.01% |
| 240 | Taste transduction | ko04742 | 1 | 0.01% |
| 241 | Type I diabetes mellitus | ko04940 | 1 | 0.01% |
| 242 | Carbohydrate digestion and absorption | ko04973 | 1 | 0.01% |
| 243 | Vitamin digestion and absorption | ko04977 | 1 | 0.01% |
| 244 | Mineral absorption | ko04978 | 1 | 0.01% |
| 245 | Prion diseases | ko05020 | 1 | 0.01% |
| 246 | African trypanosomiasis | ko05143 | 1 | 0.01% |
| 247 | Colorectal cancer | ko05210 | 1 | 0.01% |
| 248 | Renal cell carcinoma | ko05211 | 1 | 0.01% |
| 249 | Pancreatic cancer | ko05212 | 1 | 0.01% |
| 250 | Endometrial cancer | ko05213 | 1 | 0.01% |
| 251 | Chronic myeloid leukemia | ko05220 | 1 | 0.01% |
| 252 | Acute myeloid leukemia | ko05221 | 1 | 0.01% |
| 253 | Small cell lung cancer | ko05222 | 1 | 0.01% |
| 254 | Non-small cell lung cancer | ko05223 | 1 | 0.01% |
| 255 | Systemic lupus erythematosus | ko05322 | 1 | 0.01% |
| 256 | Viral myocarditis | ko05416 | 1 | 0.01% |
